# Discovery of a non-canonical TREM2-S100A9 axis that drives pro-tumorigenic macrophage polarization and sensitizes ovarian clear cell carcinoma to IL-17 blockade

**DOI:** 10.1038/s41420-026-03244-2

**Published:** 2026-07-07

**Authors:** Shi ti Zhang, Shi-mei Zhang, Fan Jiang, Hua-ju Huang, Meng-nan Long, Hao Ai, Li hong Liu

**Affiliations:** 1 Department of Obstetrics and Gynaecology, The First Affiliated Hospital of Jinzhou Medical University, Jinzhou, 121001 China; 2 Jinan University, Guangzhou, 510632 China; 3 Department of Obstetrics and Gynaecology, The Third Affiliated Hospital of Jinzhou Medical University, Jinzhou, 121001 China; 4 Liaoning Provincial Key Laboratory of Follicle Development and Reproductive Health, Jinzhou, 121001 China; 5 Department of Cardiology, The Third Affiliated Hospital of Jinzhou Medical University, Jinzhou, 121001 China

**Keywords:** Tumour immunology, Biomarkers

## Abstract

The aggressive progression and significant chemotherapy resistance of ovarian clear cell carcinoma (OCCC) are closely related to the immunosuppressive tumour microenvironment (TME), but its potential molecular drivers are still unclear. The core of this process is the M2 polarisation of tumour-associated macrophages (TAMs), which regulates the tumour-promoting microenvironment. This study reveals a new and key signal axis responsible for this pathological intercellular dialogue. We confirmed that TREM2 is highly expressed on OCCC-related TAMs and plays a key role in its M2 differentiation. In terms of mechanism, we found that TREM2 bypassed its classic signal adaptor and directly interacted with the calcium-binding protein S100A9. This unprecedented interaction acts as a molecular switch, specifically activating the IL-17 signalling pathway. As a result, the TREM2-S100A9-IL-17 cascade reaction strongly drives M2 polarisation, which in turn promotes the proliferation, migration and invasion of OCCC cells. The key is to inhibit IL-17A by gene silencing S100A9 or using the pharmacology of clinical-grade antibody secukinumab. These tumour-promoting effects are completely eliminated in both in vitro and preclinical xenotransplantation models. This study reveals a new mechanism by which macrophages drive cancer progression, and determines that the TREM2-S100A9-IL-17 axis is a key molecular weakness, which provides a strong basis for the reuse of anti-IL-17 therapy to disintegrate the OCCC immunosuppression barrier.

## Introduction

Cell fate: Whether differentiation, proliferation, survival, or apoptosis is not determined in isolation but is profoundly shaped by complex interactions with the surrounding microenvironment. In the context of cancer, this intercellular dialogue is hijacked to form a pathological microenvironment that promotes malignant tumour development. TME is a multi-dimensional ecosystem, including cancer-related fibroblasts, endothelial cells, and a variety of immune cell groups, which together promote tumour proliferation, invasion, and immune escape [1]. Among the immunosuppressive cellular components of the TME, TAMs are recognised as one of the most abundant and functionally critical cell types [2]. Macrophages, as the core builders of this microenvironment, are renowned for their remarkable phenotypic plasticity: These TAMs generally differentiate into two functional states: classically activated M1 macrophages, which mediate anti-tumour activities, and alternatively activated M2 macrophages, which drive tumour progression [3]. In tumours, the decisive polarisation of TAMs toward M2-like is a critical hallmark of aggressive diseases, directly promoting cancer cell proliferation and conferring resistance to apoptosis. However, the precise molecular switch determining this pro-tumour differentiation fate remains a key and unresolved area in cancer biology.

Ovarian clear cell carcinoma (OCCC) is a classic example of this pathological interaction, with the highest mortality rate among gynaecological malignant tumours in the world, and the five-year survival rate of advanced cases is still less than 30% [4]. OCCC is highly aggressive, characterised by dense infiltration of TAMs [5], which is closely associated with chemotherapy resistance and poor patient prognosis. In OC TME, the vast majority of TAMs show M2-like phenotypes. These M2-TAMs promote tumour growth, support angiogenesis and metastasis by secreting a variety of cytokines, chemokines and growth factors, while inhibiting cytotoxic T cell reactions and establishing an immunosuppressive microenvironment [6, 7]. Therefore, it has become a very attractive treatment strategy to reprogram these tumour-promoting M2-type TAMs into anti-tumour M1 phenotypes [8, 9].

TREM2, a transmembrane receptor of the immunoglobulin superfamily, has been confirmed as a key factor coordinating myeloid cell function, with its role involving various diseases including cancer. It is primarily expressed on myeloid cells, including macrophages, dendritic cells, and microglia [10, 11]. Through the adaptive protein DAP12, TREM2 transmits downstream signals to regulate a variety of biological processes, such as cell phagocytosis, survival, and inflammatory response [12]. Although initially recognised for its critical role in neurodegenerative disorders like Alzheimer’s [13], accumulating evidence shows its significant contribution to tumour immunity [14]. Notably, TREM2 is highly expressed in TAMs in a variety of malignant tumours, including glioblastoma, lung cancer and colorectal cancer, and it is involved in maintaining the immunosuppressive phenotype of these tumours [15, 16]. Furthermore, single-cell RNA-seq has identified TREM2⁺ TAMs as a distinct pro-tumour cell subpopulation across several tumour models [17].

Although it is known that TREM2 can affect macrophage polarisation, its polarisation type and downstream signal transduction mechanism in ovarian clear cell carcinoma have not been fully clarified. The classic TREM2 pathway relies on connecting proteins such as DAP12, but whether this single axis can explain all the situational-dependent functions of TREM2 is increasingly being questioned. This knowledge gap raises a key question: what kind of molecular mechanism links the activation of TREM2 on TAMs with the malignant proliferation and survival of OCCC cells? We speculate that TREM2 initiates a specific signal cascade reaction through non-classical interaction, which is customised for the OCCC microenvironment. Clarifying this pathway will not only reveal the basic mechanism of communication between cancer cells, but also discover potential therapeutic targets.

One of the hallmark features of OCCC is its close association with endometriosis; ovarian clear cell carcinoma often originates from endometriosis: a typical chronic inflammatory microenvironment rich in “injury signals,” which provides an ideal upstream context for the TREM2 activation identified in this study. The selection of the ES2 cell line enables us to more accurately simulate this unique pathophysiological environment of OCCC. Our findings reveal that the TREM2-S100A9-IL-17 axis essentially functions as an inflammatory amplifier, aligning perfectly with the “inflammation-driven” nature of ovarian clear cell carcinoma.

This study reveals a previously uncharacterized signaling pathway, which provides a direct answer to the question. For the first time, we confirmed that TREM2 has a physical interaction with calcium-binding protein S100A9 by bypassing the classical binding protein. As a specific molecular trigger, this interaction activates the IL-17 signaling pathway in macrophages and drives them to differentiate into the M2 type with the ability to promote tumor generation. The resulting macrophage-derived IL-17 can directly act on OCCC cells and promote their proliferation and survival. The key is that we prove the signal axis is therapeutically feasible: pharmacological blocking of the clinical-grade antibody secukinumab can effectively inhibit tumor growth.

Ovarian clear cell carcinoma exhibits high aggressiveness and significant resistance to platinum-based chemotherapy, with a markedly poorer prognosis compared to common serous ovarian cancer, making it one of the most lethal subtypes of gynecological malignancies worldwide. This study clarifies a complete and novel signal cascade response to regulate macrophage differentiation and cancer cell proliferation, providing a new mechanism framework and promising treatment strategies for OCCC. By focusing on specific clinical subtypes, we aim to elucidate the unique pathogenic mechanisms and potential therapeutic targets in OCCC, addressing urgent clinical needs.

## Results

### High expression of TREM2 in OC correlates with poor prognosis of OC

**Fig. 1:**
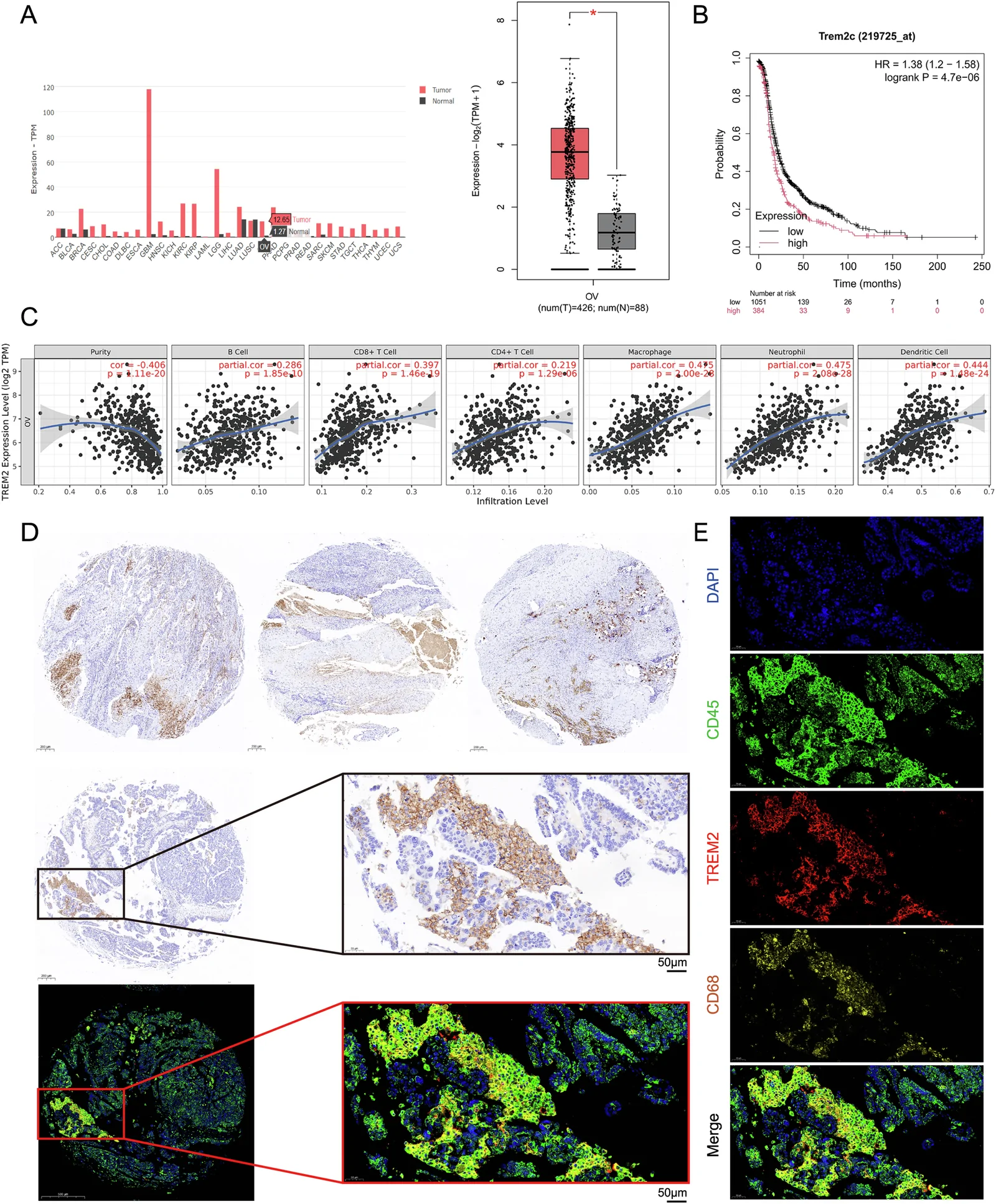
Elevated TREM2 expression is associated with poor prognosis in ovarian cancer. **A** GEPIA analysis of TREM2 mRNA expression in 426 ovarian cancer (OC) samples and 88 normal ovarian tissue samples from the TCGA database (log₂FC = 2.588, *P* < 0.001). **B** Kaplan–Meier survival analysis illustrating the association between TREM2 expression and overall survival in 1,435 patients with ovarian cancer (HR = 1.38, *P* < 0.001). **C** TIMER analysis showing the correlation between TREM2 expression and immune cell infiltration levels in ovarian cancer (*P* < 0.001). **D** Tissue microarray (diameter 1.5 mm) representative IHC staining images showing TREM2 expression in human ovarian cancer tissues. **E** Immunofluorescence co-staining of ovarian cancer tissues showing the localization of TREM2 and immune cell markers. Tissue sections were stained with antibodies against CD45 (Alexa Fluor 488, green), TREM2 (Cy3, red), and CD68 (Cy5, orange), with nuclei counterstained using DAPI (blue).

Analysis of the TCGA (The Cancer Genome Atlas) database via the GEPIA platform revealed that TREM2 is significantly elevated across various tumor types. Specifically, TREM2 mRNA expression was strikingly higher in 426 ovarian cancer (OC) tissue samples versus 88 normal ovarian tissue samples (log2FC = 2.588, *P* < 0.001; GEPIA: Fig. 1A).

The Kaplan-Meier plotter database was employed to evaluate the correlation between TREM2 expression and clinical outcomes in 1435 OC patients. Patients with high TREM2 expression demonstrated significantly poorer overall survival compared to those with low expression (HR = 1.38, *P* < 0.001, Fig. 1B).

### Increased TREM2 expression in OC-associated TAMs and its role in promoting M2 polarisation

By evaluating the correlation between TREM2 expression and immune cell infiltration in OC with TIMER, we found that TREM2 expression was significantly positively related to the infiltration level of a variety of immune cells (especially macrophages, neutrophils and dendritic cells) (*P* < 0.001, Fig. 1C).

**Fig. 2:**
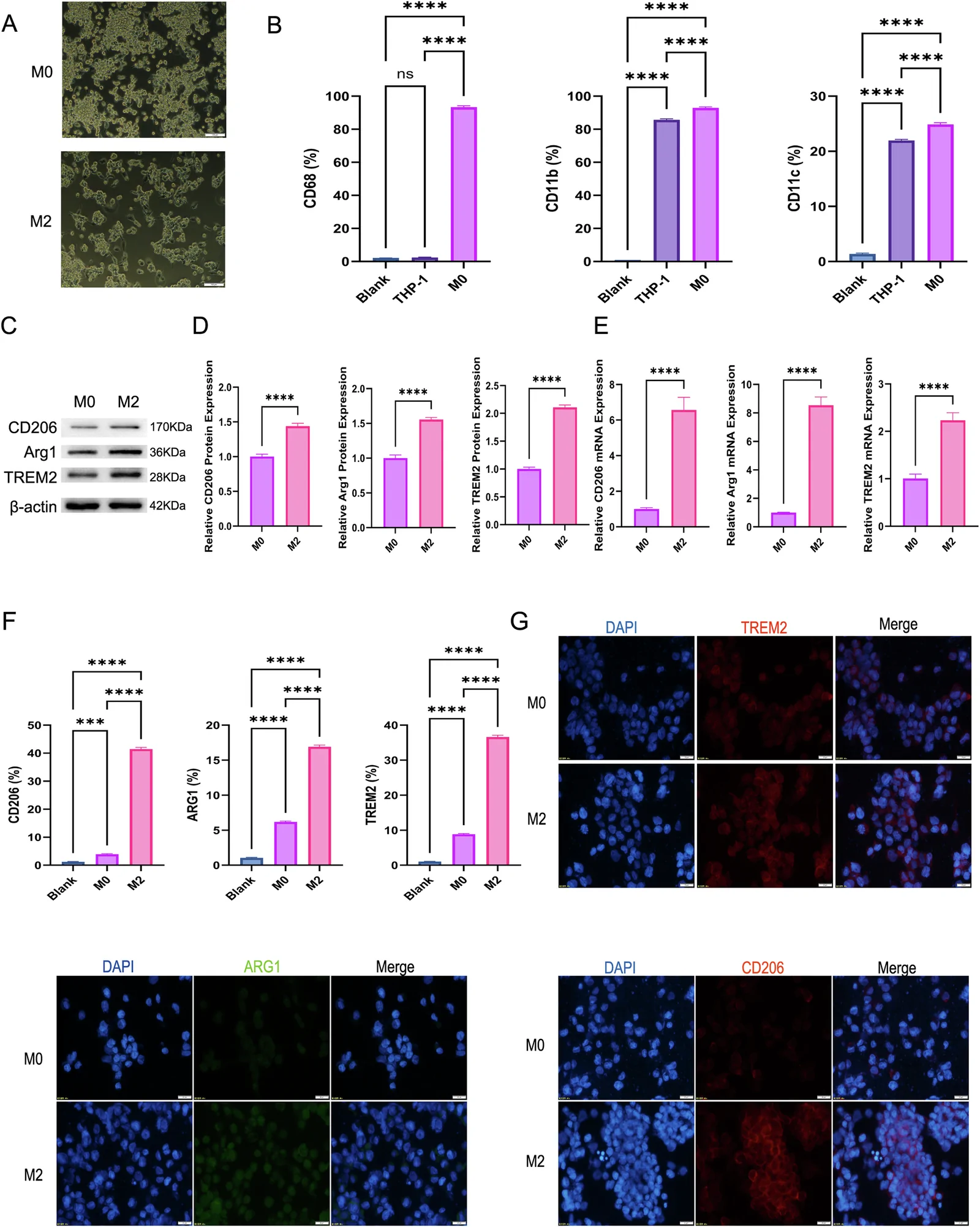
Tumor-conditioned medium promotes TREM2 expression and induces M2 polarization of macrophages (THP-1). **A** Morphological characterization of THP-1–derived macrophages. THP-1 cells were differentiated into M0 macrophages by PMA treatment, followed by exposure to ovarian cancer cell–conditioned medium to induce M2 polarization. Representative images show morphological changes during macrophage differentiation. **B** Flow cytometric analysis of macrophage surface markers. Expression levels of CD11c, CD11b, and CD68 were assessed in THP-1 and M0 macrophages following treatment with PMA. **C** Western blot analysis of TREM2, CD206, and ARG1 protein expression in macrophages under different treatment conditions (Macrophages treated with PMA are labeled as M0, and those treated with ovarian tumor conditioned medium are labeled as M2). **D** Densitometric quantification of Western blot results of TREM2, CD206, and ARG1 in M0 and M2. **E** Quantitative real-time PCR analysis of TREM2, CD206, and ARG1 mRNA expression in M0 and M2. **F** Flow cytometric analysis of TREM2, CD206, and ARG1 expression levels in M0 and M2. **G** Immunofluorescence staining showing the expression and localization of TREM2, CD206, and ARG1 in M0 and M2. (*P < 0.05, **P < 0.01, ***P < 0.001, ****P < 0.0001, ns not significant).

To explore the clinical relevance of TREM2, we initially performed immunohistochemistry (IHC) on a human ovarian cancer Tissue Microarray (TMA) (Shanghai YBL Biotechnology Co., Ltd) (Fig. 1D). Immunofluorescence (IF) co-staining subsequently determined the primary cellular localization of TREM2, revealing its predominant expression in CD45^+^ CD68^+^ TAMs within the TME (Fig. 1E). When THP-1 macrophages were stimulated with PMA with a concentration of 150 nmol/L for 12 h, and then treated with the conditional culture medium of the OC cell line, we observed that the expression of TREM2 was significantly upregulated, and the level of M2 polarisation markers (Arg1, CD206) also increased accordingly (Fig. 2A–G). These results propose that TREM2 is markedly expressed in OC-associated TAMs and promotes M2 polarisation, with its expression likely influenced by tumour-derived signals, indicating its pro-tumorigenic potential.

### TREM2 is crucial for macrophage M2 polarisation and promotes OCCC cell proliferation

To comprehensively validate the role of TREM2 in macrophage M2 polarization, we employed both functional knockout (TREM2 knockdown) and functional gain (TREM2 overexpression) strategies.

**Fig. 3:**
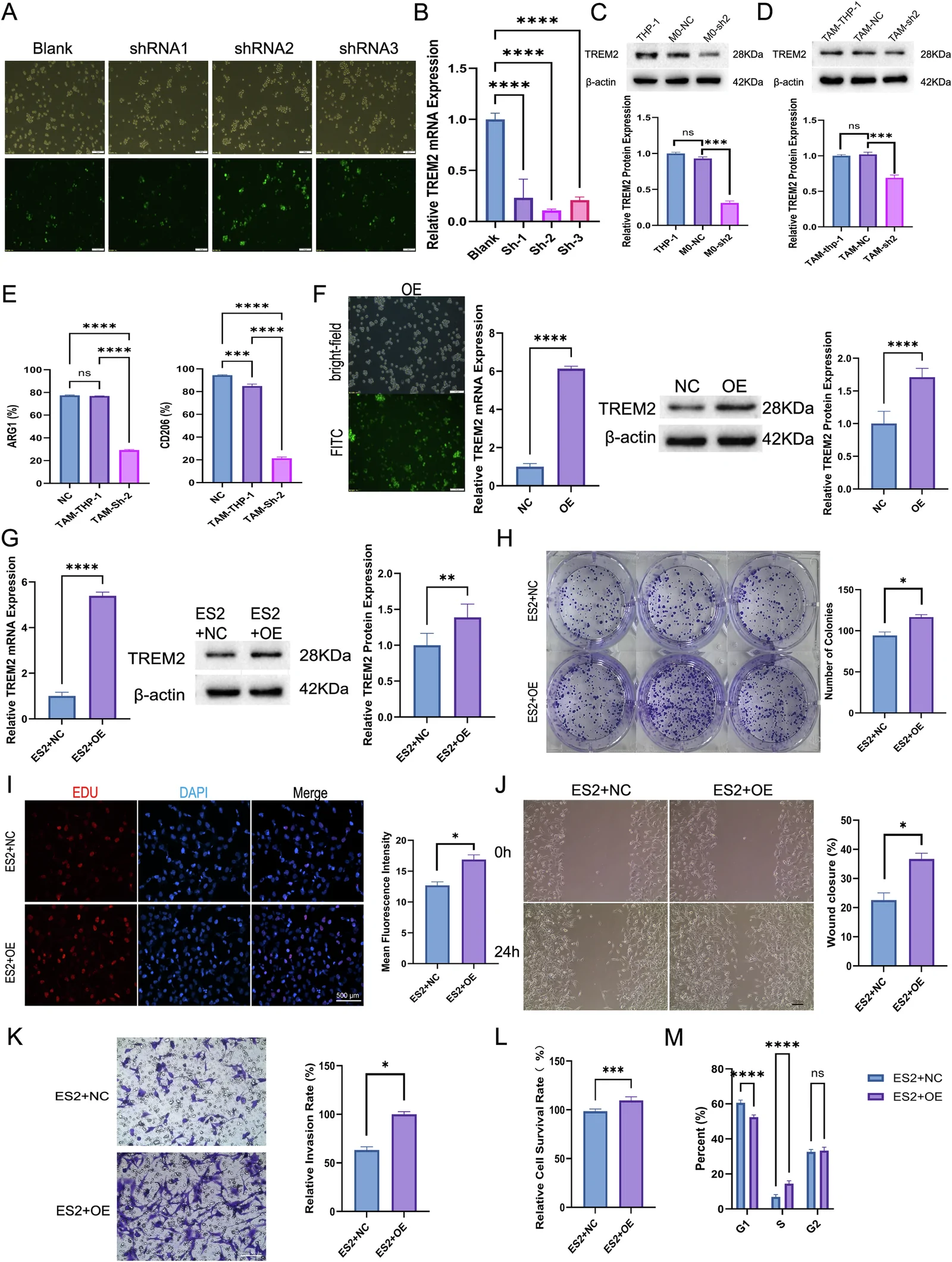
TREM2 induces macrophage (THP-1) M2 polarization and promotes the proliferation, migration, and invasion of OCCC. **A** Fluorescence microscopy images showing transfection efficiency of GFP-labeled lentiviral vectors in THP-1 cells. Green fluorescence indicates successfully transduced cells. **B** Quantitative real-time PCR analysis validating the knockdown efficiency of TREM2 in THP-1 cells; shRNA-2 (sh2) was selected for subsequent experiments. **C** Western blot analysis confirming TREM2 protein knockdown in PMA-treated THP-1 cells. Cells transfected with control shRNA were designated as M0-NC, whereas those transfected with TREM2 shRNA were labeled as M0-sh2. In (**C**), the protein expression level of TREM2 was significantly reduced in the knockdown group (PMA-treated M0-sh2) compared to the control group (PMA-treated M0-NC). **D** Western blot analysis of TREM2 expression in THP-1 derived macrophages exposed to ovarian cancer cell–conditioned medium. Cells were designated as TAM-THP-1, TAM-NC, and TAM-sh2, respectively. In (**D**), the protein expression level of TREM2 was significantly reduced in the knockdown group (TAM-sh2 treated with ovarian cancer condition culture medium) compared to the control group (TAM-NC treated with ovarian cancer condition culture medium). **E** Flow cytometric analysis of macrophage polarization markers CD206 and Arg1 in THP-1–derived macrophages following induction and treatment with ovarian cancer cell–conditioned medium. **F** Verification of TREM2 overexpression transfection efficiency under fluorescence microscope, WB, and qPCR in THP-1 cells. **G** Overexpressed thp-1 was co-cultured with ES2 cells, and the TREM2 levels were detected by PCR and Western blotting. **H** After co-culturing, assay the detection of ES2 cell clonogenic capacity by colony formation assay. **I** Detection of cell proliferation by EdU immunofluorescence assay. **J** Detection of cell migration ability by wound healing assay. **K** Detection of cell invasion ability by Transwell assay. **L** Detection of cell proliferation by CCK-8 assay. **M** Analysis of cell cycle distribution by flow cytometry. (*P < 0.05, **P < 0.01, ***P < 0.001, ****P < 0.0001, ns not significant).

In order to evaluate the key role of TREM2 in M2 macrophages, we used shRNA technology to silence the expression of TREM2 in THP-1 macrophages (Fig. 3A, B). The Western blot experiment was conducted to verify the efficiency of TREM2 knockdown under two different induction methods (PMA treatment and treatment with ovarian cancer cell condition culture medium) (Fig. 3C, D). Since conditioned culture of ovarian cancer cells significantly upregulated TREM2, in the Fig. 3D knockout group (TAM-sh2), although the bands still appeared to be expressed, the decrease was significant compared to the extremely high levels observed in the control group (TAM-NC). Our research results show that TREM2 gene knockout significantly weakened the upregulation of M2 polarisation markers Arg1 and CD206 induced by OC condition culture medium (Fig. 3E). The evidence supports that TREM2 plays a key role in promoting the polarisation of macrophages to M2.

In addition, the co-culture experiment of TREM2 overexpressed THP-1 (Fig. 3F, G) and ES2 cells found that TREM2 can significantly promote the proliferation, migration, and invasion ability of ES2 cells. Through detection methods such as EdU, colony formation, wound healing experiments, and Transwell invasion experiments, CCK8 and cell cycle analysis, the ES2 cell proliferation, migration and invasion abilities of the TREM2 overexpressed macrophage group were significantly improved compared with the control group (ES2 + NC) (Fig. 3H–M). Based on these results, TREM2 creates a tumour microenvironment conducive to the proliferation, migration and invasion of OCCC cells by promoting the polarisation of macrophages to M2.

### S100A9 is a key downstream mediator of TREM2 signal transduction

**Fig. 4:**
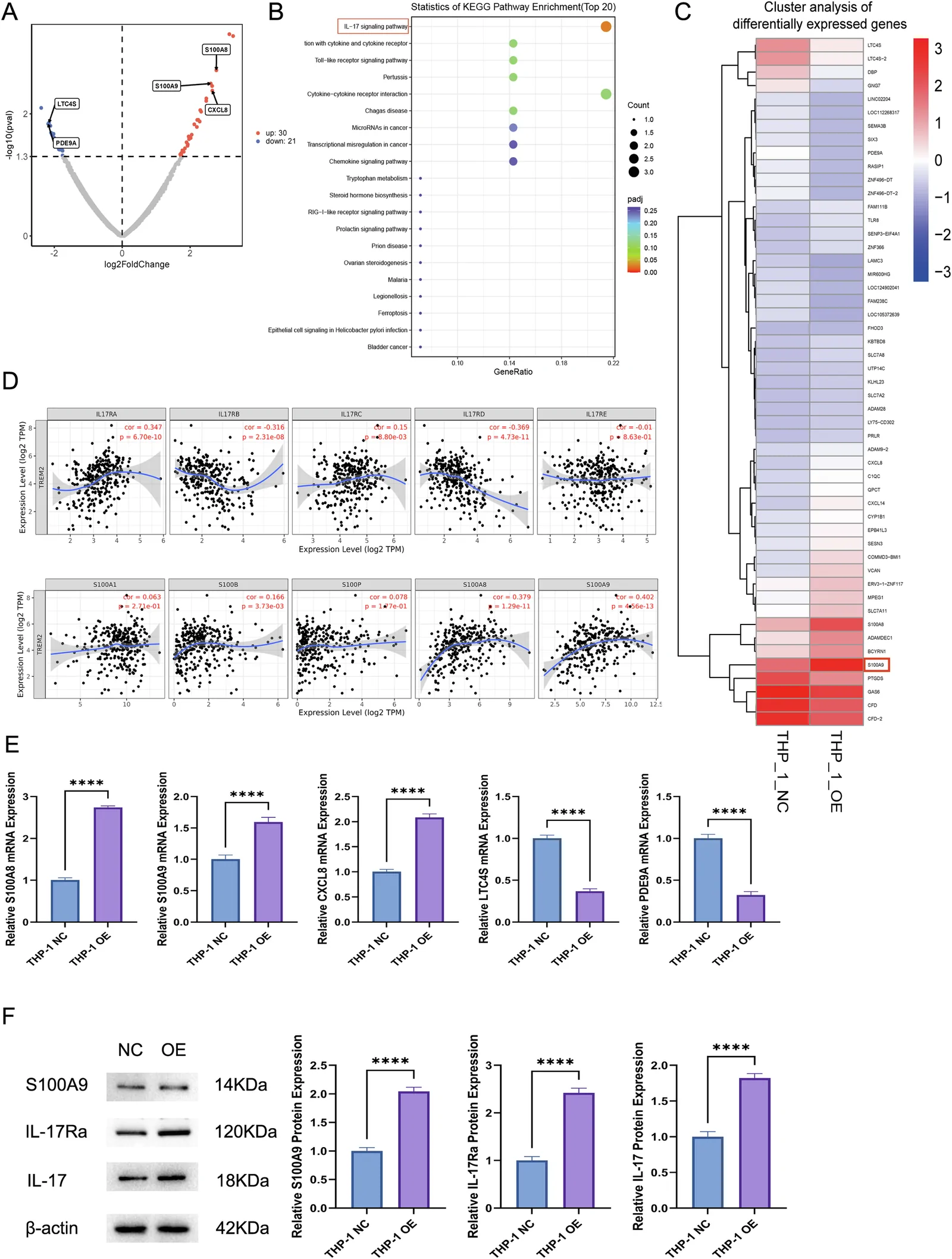
Screening of downstream genes and signal pathways regulated by TREM2. **A** DEGs associated with THP-1-NC and THP-1-TREM2-OE identified by RNA-seq. **B** KEGG pathway enrichment analysis highlighting significant enrichment of the IL-17 signaling pathway. **C** Identification of S100A9 as the most strongly associated downstream DEG of TREM2. **D** Correlation analysis using the TIMER database showing positive correlations between TREM2 and different ligands of the IL-17 pathway family and S100A protein family in ovarian cancer. The expression of TREM2 in ovarian cancer was positively correlated with IL-17RA and S100A9, which are the strongest co-expressions (*p* < 0.001). **E** Validation of selected DEGs by RT-qPCR (S100A8, S100A9, CXCL8, LTC4S, PDE9A). **F** Protein-level detection of IL-17 IL-17Ra S100A9 by WB. (* *P* < 0.05, ** *P* < 0.01, *** *P* < 0.001, **** *P* < 0.0001, ns not significant).

In order to clarify the molecular mechanism of TREM2 function, we conducted RNA sequencing analysis for THP-1 cells (THP-1 OE) and control groups (THP-1 NC) that overexpressed TREM2. Unbiased transcriptomic analysis has identified hundreds of differentially expressed genes, among which the significant upsulation of pro-inflammatory “danger signal” molecules S100A8 and S100A9 is particularly prominent (Fig. 4A). KEGG pathway enrichment analysis focused on inflammation further highlighted a strong association with the IL-17 signalling pathway [18] (Fig. 4B). Identifying S100A9 as the most strongly associated downstream-associated factor (Fig. 4C). Furthermore, data from the TIMER website indicated positive correlations between TREM2 and IL-17, IL-17RA, S100A8, and S100A9 (*P* < 0.001, Fig. 4D). We confirmed significantly increased S100A9 expression in TREM2-OE macrophages using Quantitative real-time PCR (qRT-PCR) and Western blot (WB). Concurrently, the expression and secretion levels of IL-17 and IL-17RA were markedly elevated (Fig. 4E, F).

### TREM2-S100A9-IL-17 axis regulates the pro-tumour function of macrophages

**Fig. 5:**
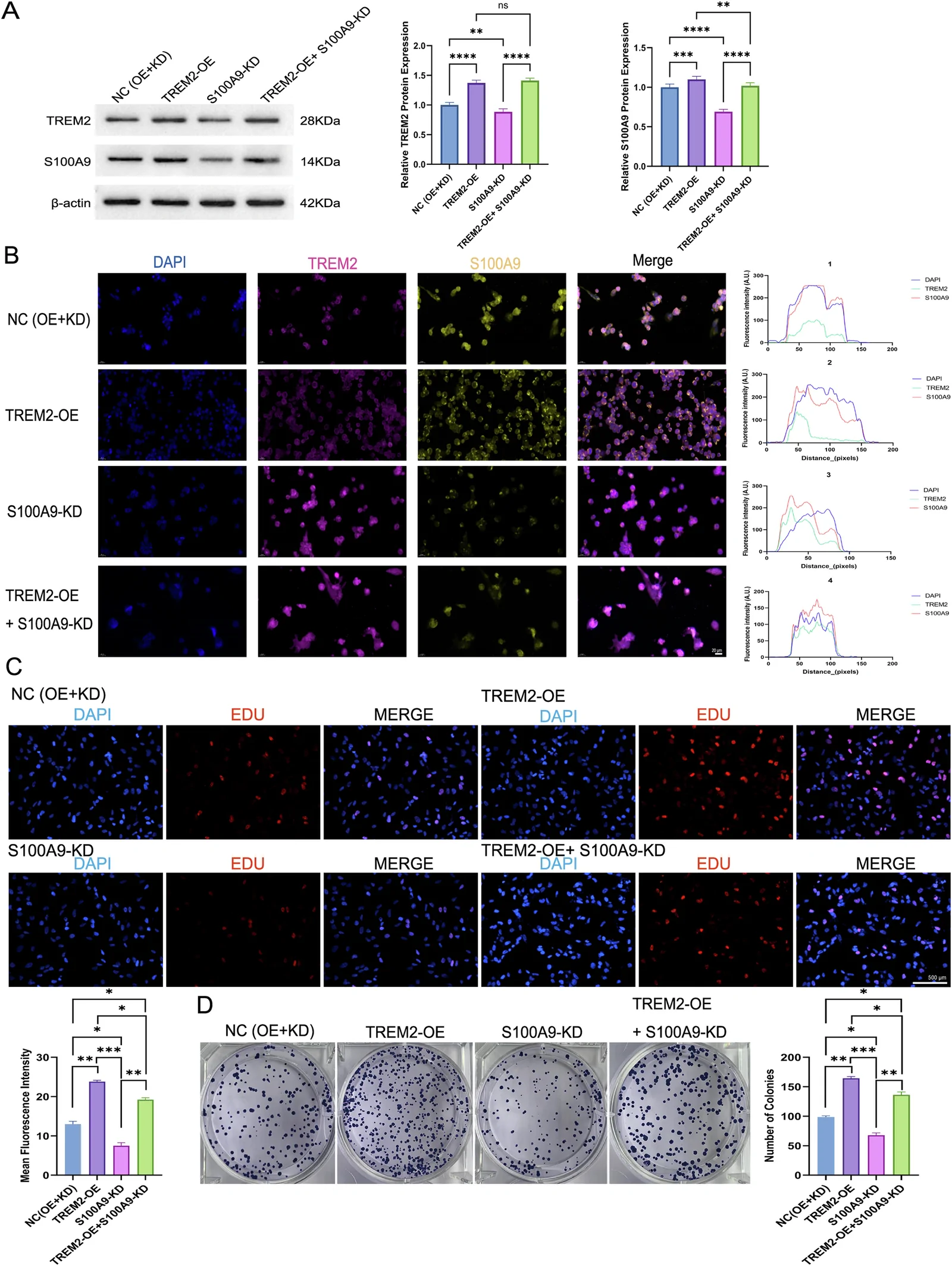
TREM2 promotes ovarian cancer progression through downstream regulation of S100A9. **A** Overexpress TREM2 and knock down S100A9 in THP-1 cells, analyze the expression of TREM2 and S100A9 detected by Western blotting. **B** Cellular localization and co-localization of TREM2 and S100A9 in THP-1 assessed by immunofluorescence staining, primarily demonstrating their spatial proximity. **C** After co-culturing, assay ES2 cell proliferation evaluated using the EdU incorporation assay. **D** Cell proliferation assessed by colony formation assay. (*P < 0.05, **P < 0.01, ***P < 0.001, ****P < 0.0001, ns not significant).

**Fig. 6:**
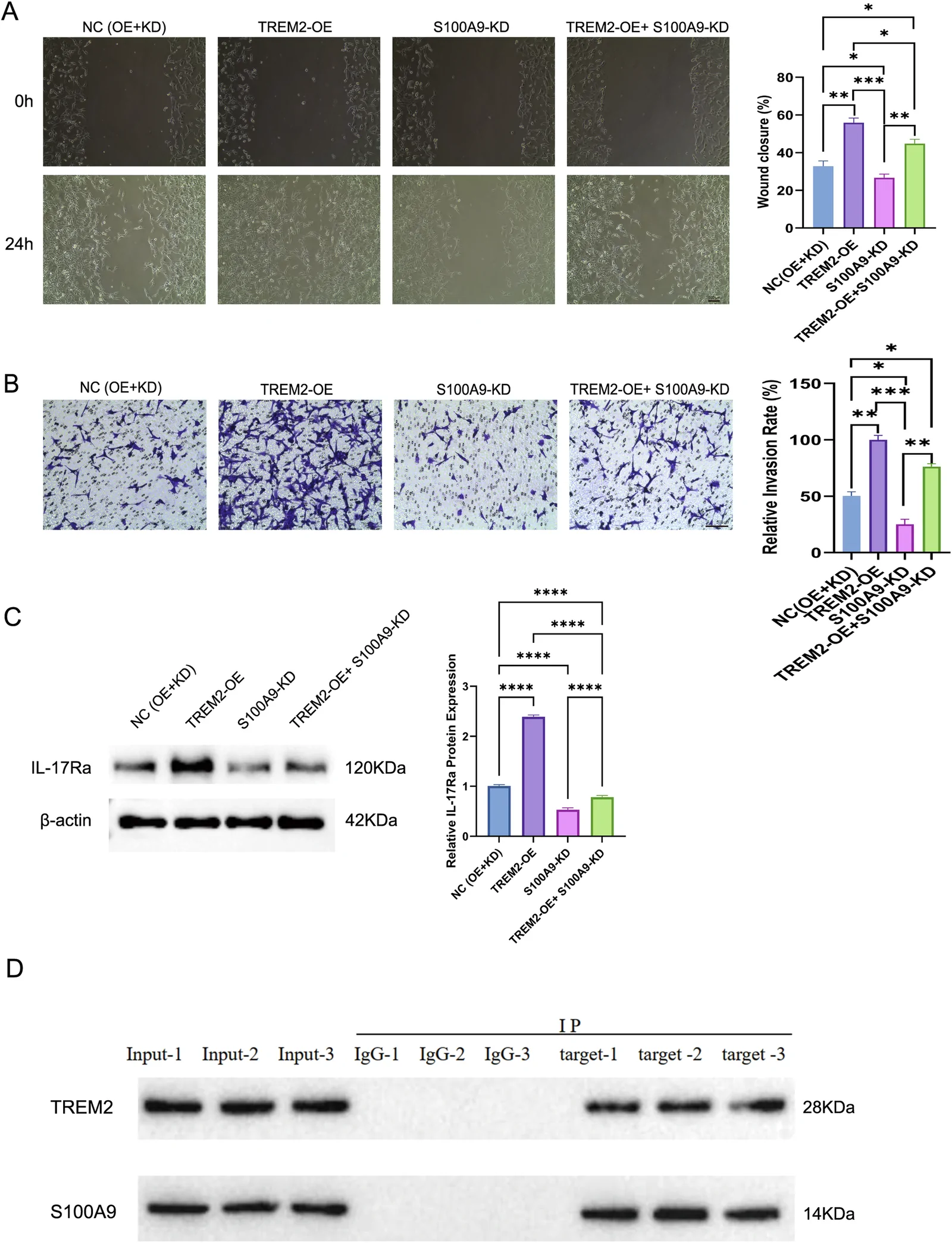
Interaction between TREM2 and S100A9. **A** Overexpress TREM2 and knock down S100A9 in THP-1 cells, co-culture it with ES2 cells, after co-culturing, assay ES2 cell migration evaluated by wound healing assay. **B** After co-culturing, assay ES2 cell invasion assessed by Transwell assay. **C** Western blotting showing that S100A9 knockdown reduces IL-17RA protein expression. **D** Interaction between TREM2 and S100A9 confirmed by co-immunoprecipitation (Co-IP). (*P < 0.05, **P < 0.01, ***P < 0.001, ****P < 0.0001, ns not significant).

To functionally validate the role of the TREM2-S100A9 axis, we performed S100A9 gene knockdown experiments. Overexpress TREM2 and knock down S100A9 in THP-1 cells, analyze the expression of TREM2 and S100A9 detected by Western blotting (Fig. 5A). Immunofluorescence analysis indicated a co-localization of TREM2 with S100A9 (Fig. 5B). Set DAPI as the blue channel, TREM2 as the green channel, and S100A9 as the red channel. In the image, the peaks and troughs of TREM2 and S100A9 are synchronized, demonstrating intracellular co-localization of these two target genes in tumor-associated macrophages and providing morphological evidence for subsequent Co-IP experiments to verify their direct protein interaction. In macrophages overexpressing TREM2 and undergoing S100A9 knockdown (TREM2-OE + S100A9-KD), the significantly enhanced proliferative (Fig. 5C, D), migratory (Fig. 6A), and invasive capabilities (Fig. 6B) of ES2 cells in co-culture were entirely suppressed. Western blot analysis further demonstrated that S100A9 silencing led to a decrease in IL-17RA expression (Fig. 6C), thereby confirming S100A9’s function as an upstream regulator in IL-17A induction. Co-immunoprecipitation (Co-IP) assays confirmed a direct protein-protein functional interaction between TREM2 and S100A9 (Fig. 6D), suggesting a direct regulatory connexion within this pathway.

**Fig. 7:**
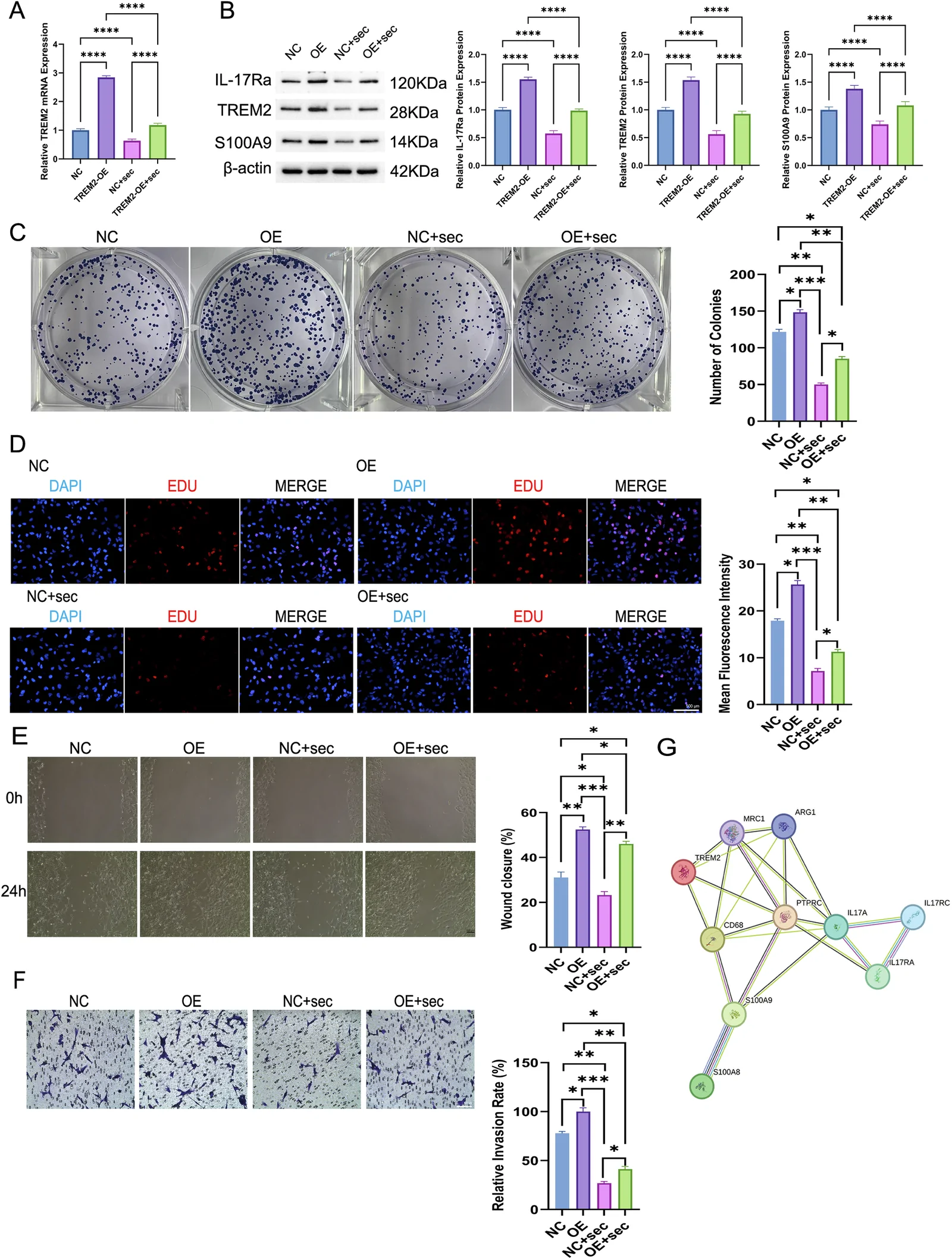
Confirmation of TREM2 regulating ovarian cancer progression via IL-17 signaling pathway. **A** Co-culture of THP-1-OE with IL-17 pathway inhibitors (Secukinumab) and ES2. Detection of TREM2 expression by RT-PCR. **B** Detection of TREM2, IL-17Ra, and S100A9 expression by WB. **C** Detection of cell proliferation by colony formation assay. **D** Detection of cell proliferation by EdU assay. **E** Detection of cell migration by wound healing assay. **F** Detection of cell invasion by Transwell assay. **G** Protein interaction network mapping using the STRING platform: interactions between TREM2 and nine other genes in this study (CD45, CD68, S100A9, S100A8, IL-17Ra, IL-17Rc, IL-17, Arg1, CD206). (*P < 0.05, **P < 0.01, ***P < 0.001, ****P < 0.0001, ns not significant).

To further explore the role of the IL-17 signalling pathway, we utilised the clinical-grade IL-17A inhibitor secukinumab (MCE, AIN457, USA) [19]. Western blot and PCR analyses showed that treatment with the inhibitor suppressed upstream factors TREM2 and S100A9, as well as downstream component IL-17Ra (Fig. 7A, B). Treatment with secukinumab significantly reversed the enhanced invasive phenotype of OC cells induced by TREM2-OE macrophage (Fig. 7C–F). Finally, we utilised the STRING platform to construct a protein–protein interaction (PPI) network (Fig. 7G) involving TREM2 and nine related genes: CD45 (PTPRC), CD68, S100A9, S100A8, IL-17Ra, IL-17Rc, IL-17, Arg1, CD206 (MRC1), which further corroborated our mechanistic findings. In conclusion, this study uncovers a novel signalling cascade where TREM2 stimulates the IL-17 pathway via its functional interaction with S100A9, thereby promoting macrophage M2 polarisation and enhancing tumour-promoting activities.

### Targeting the TREM2-S100A9 axis can inhibit the in vivo growth of OCCC

**Fig. 8:**
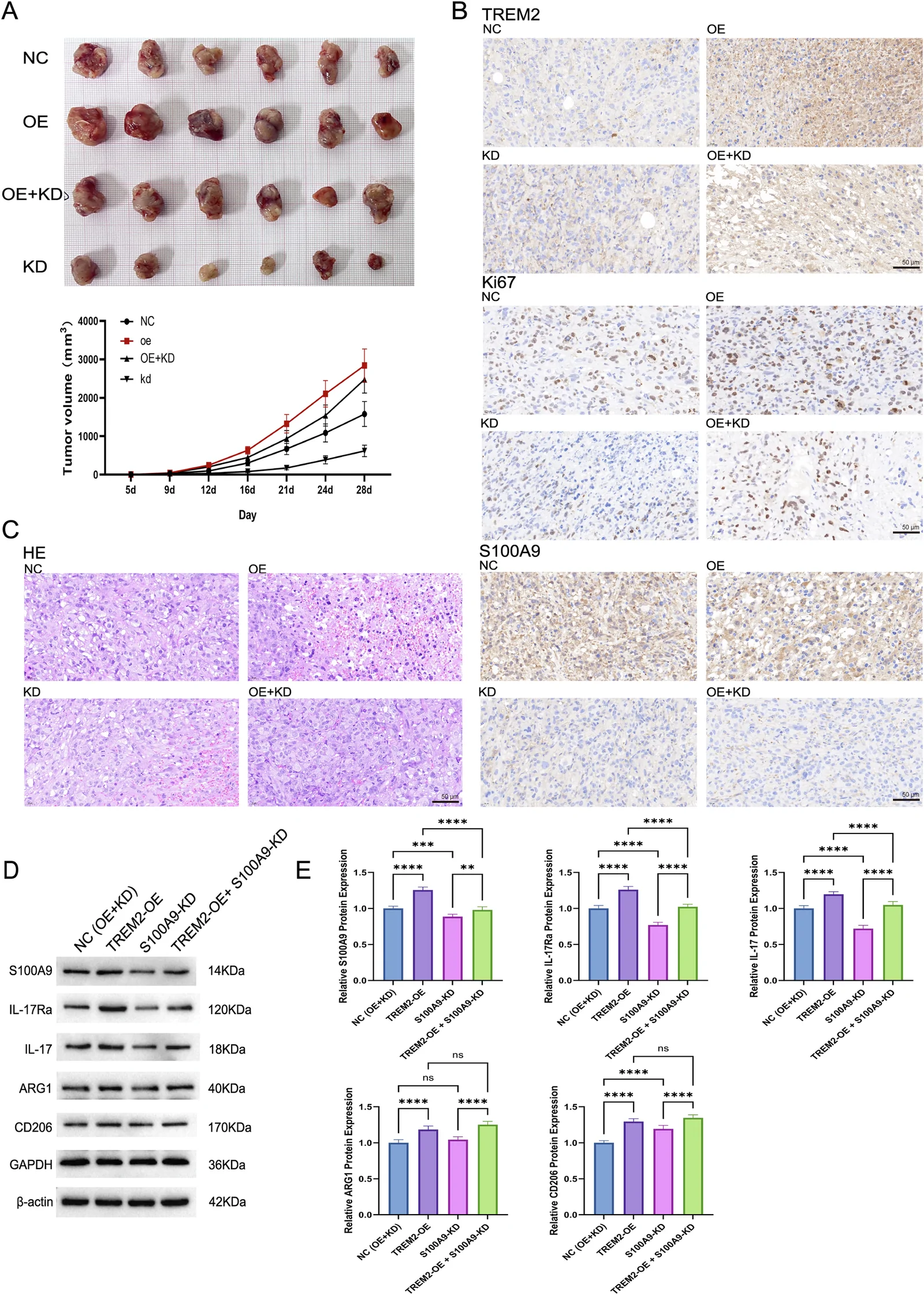
To investigate the in vivo mechanism by which TREM2 regulates ovarian cancer progression through downstream gene S100A9 expression. **A** Established a subcutaneous xenograft model in nude mice using macrophages overexpressing TREM2 and S100A9 knockdown. The gross morphology of xenograft tumors was observed, and tumor size recorded. **B** Immunohistochemical detection of TREM2, S100A9, and Ki67 expression. **C** Histopathological features of tumor tissues were examined via HE staining. **D**, **E** Western blot analysis was performed to detect expression of IL-17 signaling pathway-associated proteins. (*P < 0.05, **P < 0.01, ***P < 0.001, ****P < 0.0001, ns not significant).

To corroborate our in vitro findings within a physiological context, we established a subcutaneous xenograft model in nude mice. Co-injection of TREM2-overexpressing macrophages with ES2 cells markedly expedited tumour growth, resulting in final tumour volumes and weights that were markedly greater than the control group. Crucially, the robust pro-tumour effect was completely abolished when S100A9 was concurrently silenced in the TREM2-OE macrophages (TREM2-OE + S100A9-KD) (Fig. 8A). IHC analysis of the excised tumours tissues indicated considerably elevated levels of the proliferation markers Ki67 and S100A9 within the TREM2-OE group; levels were restored to baseline in the TREM2-OE + S100A9-KD cohort (Fig. 8B). Furthermore, histopathological examination (H&E staining) revealed pronounced nuclear heterogeneity in TREM2-OE tumour cells compared to the S100A9-KD group, indicative of a higher degree of malignancy (Fig. 8C). Western blot analysis of homogenised tumour lysates further confirmed that the protein expression profiles of TREM2, S100A9, IL-17, IL-17Ra, Arg1, and CD206 were consistent with our in vitro results (Fig. 8D, E). These robust in vivo findings unequivocally affirm the TREM2-S100A9-IL-17 axis as a crucial signalling pathway and therapeutically relevant signalling pathway that drives clear cell tumour of ovary progression.

## Discussion

### The advantage and innovation of the TREM2-S100A9-IL-17 signalling axis in ovarian cancer

TAMs to overcome immune-inhibitory TMEs represents a frontier in contemporary cancer therapy [20]. The TREM2 has been established as a pivotal regulator within the tumour immune microenvironment, significantly influencing tumour progression by modulating TAM functions. We observed that the high expression of TREM2 in OC TAMs was associated with adverse clinical outcomes, which was consistent with the reported results of a variety of solid tumours [16, 21–25]. This further confirms the potential of TREM2 as a phenotypic marker of increased malignancy and immunosuppressive TAMs. However, the TREM2 signalling pathway itself is highly plastic, and its downstream pathways show significant context specificity and organisational specificity diversity [26].

We found a new type of TREM2-S100A9-IL-17 signalling axis in ovarian clear cell carcinoma, which is different from the traditional TREM2 pathway described in other tumour contexts [27]. The traditional TREM2-Syk axis mainly regulates the survival, proliferation, phagocytic activity and secretion of various immunosuppressive cytokines of TAMs (e.g., IL-10, TGF-β) and pro-angiogenic (e.g., VEGF) factors, typically relying on DAP12/Syk as the main adaptor. In contrast, our study revealed an unusual and highly tissue-specific mechanism in OCCC TME: the direct protein-protein functional interaction between TREM2 and S100A9, which specifically activates the downstream IL-17 inflammatory signalling cascading reaction. Although the IL-17 pathway is traditionally regarded as a pro-inflammatory pathway, its role in promoting tumours through mechanisms such as angiogenesis and neutrophil recruitment is increasingly recognised [28, 29]. S100A9 is a significant damage-associated molecular pattern (DAMP) molecule, exhibits potent inflammatory and immunomodulatory activities upon engagement [30–37]. Against this background, the unique activation mode of S100A9 seems to be closely related to the unique cytokine microenvironment in OCCC TME, which may include components such as abdominal fluid and local high concentrations of S100A9. This highlights the high situational dependence of TREM2 signal transmission. Under the stimulation of TME, TAMs with high expression of TREM2 will bind the cytoplasmic calcium-binding protein S100A9 when activated, thus amplifying the IL-17 signalling pathway. This combination directly triggers the key downstream receptor IL-17Ra, effectively bypasses the needs of the traditional DAP10-Syk pathway, and accurately guides the transformation of TAMs to M2 to promote tumour. This signal amplification ultimately enhances the proliferation, migration, and invasion ability of OCCC cells, thus promoting tumour progression. Our research provides a new perspective on the pathogenesis of OCCC by directly linking the innate immune receptor (TREM2) to the powerful amplification of the specific cytokine axis (IL-17).

This mechanism suggests a highly targeted treatment route. For example, compared with extensive inhibition of Syk or DAP12, strategies that focus on destroying the functional interaction of TREM2/S100A9 or inhibiting the IL-17 pathway may have better efficacy and lower systemic toxicity, providing precise molecular intervention targets for clinicians.

### Clinical translational significance of signal axis

The TREM2-S100A9-IL-17 axis found in this study not only deepens our understanding of the mechanism of ovarian clear cell cancer progression, but more importantly, it provides multiple promising entry points for potential clinical transformation.

#### Potential prognostic biomarkers

The main members of this axon system can be used as a new candidate biomarker combination to detect tumour tissue and ascites. By using IHC to evaluate the density of TREM2^+^ TAMs in infiltrated tumour tissue, or using ELISA and spectral analysis techniques to quantify the level of soluble TREM2 or S100A8/A9 complex in the patient’s ascites and potential peripheral blood, these markers can assist in assessing the risk stratification of prognosis. Individuals with elevated levels of these markers may be associated with increased invasiveness, increased recurrence probability, and shorter overall survival. Future applications may involve identifying patient groups suitable for axial targeted treatment strategies by evaluating the concentration of biomarkers, thus promoting the development of precision oncology.

#### As potential novel therapeutic targets

##### Targeting TREM2

The development of specific antibodies or small molecule inhibitors to block TREM2 activation, or strategies aimed at selectively depleting cancer-promoting TAMs with high TREM2 expression.

##### Targeting S100A9

Small molecule inhibitors such as Paquinimod and Tasquinimod have been developed to modulate S100A9 function in other diseases [38]. Our study provides theoretical rationale for the potential repurposing of these drugs in ovarian cancer.

##### Targeting the TREM2-S100A9 interaction

The development of peptides or small molecules that specifically block the direct functional interaction between the intracellular domain of TREM2 and S100A9 may represent a more precise intervention and could potentially offer improved safety profiles compared with direct inhibition of the full function of TREM2 or S100A9.

##### Targeting the IL-17 pathway

Mature monoclonal antibodies targeting IL-17 (e.g., secukinumab) [19] or IL-17R (e.g., brodalumab) [39] have been approved for treating autoimmune diseases such as psoriasis. The potential repositioning of these drugs for OCCC may shorten the drug development cycle and reduce associated costs.

#### Potential for combination therapies

##### Combination immune checkpoint inhibitors (ICIs)

Ovarian cancer is generally regarded as an “immunologically cold” neoplasm, exhibiting low response rates to PD-1/PD-L1 therapies [40]. By interfering with the TREM2-S100A9-IL-17 pathway, TME may be functionally reprogrammed, which may promote the transformation of immunosuppressive M2 TAMs to anti-tumour M1 phenotype. This transformation can promote the transformation of “cold” tumours into a “hotter” immune microenvironment, thus enhancing the response to ICIs.

##### Combined chemotherapy alongside PARP inhibitors

There is substantial evidence indicating that TAMs are significantly associated with resistance against chemotherapy and PARP inhibitors [41–43]. By inhibiting this signalling pathway and weakening the “protective” effect of TAMs on malignant cells, the sensitivity of tumours to conventional chemotherapy or targeted therapy may be partially restored.

### Analysis of association with ovarian cancer subtypes

#### Inflammatory origin and tumour progression associated with OCCC

This study adopts the ES2 cell line, which is a recognised model of highly invasive and resistant to chemotherapy-resistant transparent cell ovarian cancer. Although the ES2 cell line does not represent the broad spectrum of all ovarian cancers, it serves as a well-established OCCC model, making it highly suitable for this study to thoroughly investigate the unique and refractory molecular mechanisms of OCCC and to develop targeted therapeutic strategies for this subtype. The strong verification found in the key model highlights the significant clinical correlation of the targeted TREM2-S100A9-IL-17 signaling pathway treatment of this special intractable ovarian cancer subtype. A key feature of OCCC is the microenvironment of OCCC exhibits a denser infiltration of macrophages [5]. Simultaneously, it has the strong association with endometriosis [44]. Endometriosis is a chronic inflammatory disease characterised by repeated bleeding, iron accumulation and persistent oxidative stress. This environment rich in “damage signals” provides an ideal upstream context for the signalling pathway identified in this study.

##### Inherent activation landscape of TREM2

Frequent bleeding leads to excessive deposition of cell fragments, apoptotic cells, and heme and iron, which provides a large number of endogenous ligands for TREM2 on the surface of macrophages. Therefore, the activation of TREM2 may represent an early and continuous event in OCCC TME, which is the key mechanism for macrophages to cope with this long-term damage environment.

##### S100A9 as an inflammatory amplifier

Under the framework of chronic inflammation and oxidative stress, the activation of TREM2 is related to the induction of S100A9 expression, thus forming a positive feedback amplification loop. This process converts the upstream damage signal recognised by TREM2 into downstream enhanced inflammation signals mediated by S100A9.

##### IL-17 signalling aligns with the inflammatory nature of OCCCs

As a key cytokine that drives strong inflammatory reactions, IL-17 is integrated into the signal cascade identified in this study. This mechanism conforms to the “inflammation-driven” cancer characteristics of OCCCs. Our research shows a unique molecular mechanism that clarifies how OCCCs use their intrinsic inflammatory microenvironment to promote proliferation, migration, and aggressiveness.

#### Major clinical challenges in interpreting OCCC—chemotherapy resistance

OCCCs are widely known for their inherent resistance to standard platinum chemotherapy regimens, which leads to a significant deterioration in the prognosis of advanced patients. TAMs are often considered to be a key factor in chemotherapy resistance. Our research reveals a potential mechanism: M2 polarisation TAMs driven by the TREM2-S100A9-IL-17 axis not only promotes the growth of tumour cells, but also may form a “protective microenvironment” for OCCCs by secreting multiple factors or promoting direct intercellular interaction. Thus, reducing the efficacy of chemotherapy drugs. Therefore, targeting this signalling pathway (for example, through S100A9 inhibitors or anti-IL-17 antibodies) can not only inhibit tumour growth, but also may enhance chemotherapy sensitivity, providing a feasible strategy for solving OCCCs drug resistance.

### Advantages and limitations of selecting THP-1 as a cell line for in vitro and in vivo experimental studies

This research primarily employed the PMA-differentiated THP-1 cell line as an in vitro model for studying TAMs, a decision made after careful methodological consideration. As a widely used, mature, and reliable macrophage function and signalling pathway detection model, the THP-1 cell line provides crucial technical advantages for this study.

In this study, we employed both TREM2 knockdown and overexpression strategies to comprehensively elucidate the role of TREM2 in the progression of OCCC. However, the choice of overexpression in functional enhancement experiments was based on the following considerations:

1. Simulation of dynamic tumor upregulation: Although TREM2 is expressed in macrophages, our study (Fig. 2) demonstrated that TREM2 expression was significantly upregulated under conditions of ovarian cancer cell-conditioned medium stimulation, accompanied by an increase in M2 polarization markers. Therefore, the use of TREM2 overexpression (TREM2-OE) in subsequent functional and in vivo experiments aimed to more accurately simulate the pathologically upregulated state of TREM2 in the tumor microenvironment, replicating the extreme phenotype of TAMs activated by tumor signals under pathological conditions. This approach enhanced signal activation of the proposed non-classical signaling pathway, thereby providing clearer and more reliable insights into its downstream mechanisms and pro-tumor functions.
2. Confirmation of the “molecular switch” role: The knockdown experiments (Fig. 3) demonstrated that TREM2 is a prerequisite for M2 polarization, highlighting its critical role in promoting macrophage M2 polarization and establishing its necessity in OCCC-associated macrophage M2 polarization. Subsequent functional experiments with overexpression further confirmed TREM2 as a sufficient driver of tumor progression.
3. Elucidation of the mechanism: To clarify the specific molecular mechanism of the TREM2-S100A9-IL-17 signaling axis, we utilized TREM2 overexpression to enhance pathway activity. TREM2 overexpression significantly enhanced the activation of the S100A9 and IL-17 signaling pathways (Fig. 4), enabling us to clearly identify S100A9 as a key downstream mediator through RNA sequencing and subsequent validation.
4. Functional validation and causality: In the functional in vitro co-culture and in vivo xenograft models depicted (Figs. 5–8), we further reduced S100A9 expression under conditions of TREM2 overexpression. This combined overexpression and knockdown experimental design allowed us to directly demonstrate that S100A9 is the critical downstream effector molecule mediating TREM2’s pro-tumor effects, and that inhibition of S100A9 completely reversed the pro-tumor effects induced by TREM2 overexpression, providing robust evidence of causality.
5. In vivo model requirements: In the xenograft model, TREM2-OE macrophages exhibited more pronounced pro-tumor effects, thereby more clearly demonstrating the reversal effect following downstream S100A9 knockdown. TREM2 overexpression provided a more intuitive illustration of how enhanced TREM2 activity directly improves the proliferation, migration, and invasion capabilities of OCCC cells, thereby confirming TREM2’s gain-of-function role in OCCC progression.

#### Consistency and reproducibility

As a genetically uniform and immortalised human mononuclear cell line, the THP-1 cell line can continue to maintain a relatively stable macrophage phenotype after differentiation. This inherent uniformity greatly reduces experimental variability, thus ensuring high reliability and repeatability in complex operations including RNA sequencing and protein function evaluation. This robustness is crucial for revealing new signal transmission mechanisms (such as the TREM2-S100A9-IL-17 pathway), which need to be clarified through detailed and systematic research.

#### Efficiency of genetic manipulation

In the mechanism verification of this study, we adopted stable transgenic overexpression (TREM2-OE) and gene silencing technology (sh-TREM2, S100A9-KD). THP-1 cells show strong tolerance and relatively high efficiency in gene manipulation methods (including slow virus transduction), which facilitates the generation of these key tool cell models. In contrast, primary macrophages, especially TAMs from the tumour microenvironment, not only have difficulties with large-scale acquisition, but also show significant resistance to exogenous gene delivery. Such cells are usually less infected and prone to cell death or unexpected phenotypic changes.

#### Scalability and standardization

The extensibility of the THP-1 cell culture system facilitates experiments that require a large number of cell materials (including RNA-seq, CO-IP, WB). In addition, the recognised scheme of PMA-induced THP-1 cell differentiation has achieved standardised experimental conditions, so that our research results can be directly compared and integrated with the existing research using this model.

#### Mechanism clarification

The important contribution of this study is to identify the signalling pathway of TREM2-S100A9-IL-17, revealing its direct relationship with the increase in the malignancy of ovarian cancer. By adopting the efficient THP-1 cell line, we have successfully reduced the influence of mixed variables, thus clearly clarifying the causal relationship between the functional interaction of TREM2-S100A9 and the activation of the IL-17 signalling pathway. This task, used in highly heterogeneous primary TAMs, is still challenging. The discovery marks a mechanical breakthrough of great scientific significance.

However, one of the limitations of this study lies in the way the conclusion is interpreted: it should not be regarded as the TREM2-S100A9-IL-17 axis is an important pathway for TAMs to play an important role in ovarian cancer. In the actual TME, this newly discovered pathway is likely to coexist with traditional immunosuppressive pathways such as IL-10/TGF-β, and angiogenic pathways such as VEGF. The THP-1 model may amplify the role of the IL-17 pathway and underestimate the influence of other signalling pathways. Therefore, when considering the transformation of these findings into treatment strategies, it is necessary to evaluate whether the single inhibition of the IL-17 pathway is sufficient to significantly weaken the tumour-promoting function of TAMs in the patient’s body, or whether it is necessary to target other pathways in combination.

Future research should focus on complex and physiologically relevant models, such as TAMs derived from primary patients, organ-like co-culture systems or human-derived mouse models, to verify the activation status of the TREM2-S100A9-IL-17 axis, and further clarify the IL-17 environment-dependent molecular characteristics of the pathway. This assessment will help assess its relative role and therapeutic potential in a broader network of TAMs carcinogens, which requires further biochemical and structural biology research to clarify.

In summary, although our study has clearly revealed the new TREM2-S100A9-IL-17 signalling pathway in the THP-1 model, its real clinical value lies in its potential as an important biomarker and potential therapeutic target. Subsequent research should be committed to verifying the pathway in an environment with more clinical application value, especially through multi-group analysis technology to systematically study its expression characteristics and biological functions in different ovarian cancer subtypes. This strategy will eventually lay the foundation for the development of molecular stratification and individualised treatment plans.

## Conclusions

Our study found a previously ignored TREM2-S100A9-IL-17 signalling axis, which promotes the progression of OCCC by promoting the polarisation of tumour macrophages to the M2 phenotype. Our research results show that the activation of TREM2 in TAMs is associated with inflammatory amplification, and tumour growth is supported by non-classical signalling mechanisms in the TREM2 pathway.

We reveal the environmental dependence and plasticity of macrophage signal transmission, and clarify how inflammatory signals interfere with the progression of ovarian malignant tumours in the OC TME. At the same time, it is proposed that the TREM2-S100A9-IL-17 axis is a potential therapeutic weakness, providing an opportunity for biomarker development and targeted intervention.

Although further validation in clinically relevant models is required, our findings provide a mechanistic framework for understanding macrophage-driven tumour progression and support the rationale for exploring immune-modulatory strategies in OCCC. Notably, although this study focuses on macrophage-driven mechanisms, the contribution of other immune compartments within the tumour microenvironment cannot be excluded.

## Materials and methods

### Clinical samples and database analysis

#### Clinical sample source

The ovarian cancer tissue microarray (China, Shanghai; YBLBIO; YP-OvaSur2201) contains 94 cancer tissue specimens, including pathological grade and TNM staging data, treatment regimens, and survival periods.

#### Bioinformatics analysis

##### GEPIA

TREM2 RNA expression and corresponding clinical data were extracted from 514 cases of the OC dataset of the The Cancer Genome Atlas (TCGA), including 426 OC tissue samples and 88 normal ovarian tissue samples. GEPIA was used to analyse the basal TREM2 expression levels in OC and its association with clinical pathological stage.

##### Kaplan–Meier Plotter

The Kaplan–Meier Plotter database was used to generate the survival curve for OC patients stratified by TREM2 expression levels.

##### TIMER

The TIMER database was utilised to evaluate immune cell infiltration associated with TREM2 expression, as well as correlation between TREM2 and IL17, IL17Ra-f, S100A8, and S100A9.

##### STRING

The STRING database was used to construct the protein–protein interaction network of TREM2 and CD45 (PTPRC), CD68, S100A9, S100A8, IL-17Ra, IL-17Rc, IL-17, Arg1, and CD206 (MRC1).

#### RNA-seq

Total RNA was extracted from THP-1-NC and THP-1-TREM2-OE cells, purified, and subjected to fragment selection. After PCR amplification, libraries were constructed and sequenced following quality control. Differentially expressed genes (DEGs) were identified through gene expression analysis, with statistically significant genes selected using a *P* < 0.05 threshold. Subsequent analyses included Gene Ontology (GO) functional enrichment and Kyoto Encyclopaedia of Genes and Genomes (KEGG) pathway enrichment analyses, which were subsequently performed to characterise differential expression patterns.

### Cell lines and culture conditions

#### Cell lines

ES2 and THP-1 cells (China, Shanghai, Mingjin Cell Biology; originally obtained from the American Type Culture Collection, ATCC) were cultured in DMEM or RPMI-1640 medium (Procell) supplemented with 10% foetal bovine serum (Gibco) and 1% penicillin/streptomycin. Cells were maintained in a humidified incubator at 37 °C with 5% CO_2_ and passaged no more than four times before use in subsequent experiments.

#### Macrophage induction and co-culture

THP-1 cells were cultured overnight, followed by treatment with 150 nmol/L PMA (MCE, HY-18739) to induce macrophage differentiation for 12 h. Differentiated THP-1 cells (1 × 106 cells/well) were inoculated at the bottom of a 6-well transwell plate, while ES2 cells were placed in the upper chamber and co-cultured for 48 h. Macrophages in the lower chamber were then harvested for analysis. All macrophage-related genetic manipulation experiments (including TREM2 knockdown/overexpression and co-culture experiments) were performed using THP-1 cells.

#### Lentiviral transduction

Lentiviral vectors labelled with luciferase and puromycin resistance (Jima GenePharma, Shanghai, China) were used for TREM2 silencing, TREM2 overexpression, and S100A9 knockdown in THP-1 cells. After 72 h of transduction, stable cell lines selected using 2 μg/mL puromycin and collected for subsequent experiments.

### Animal model construction

#### Animals

All animals were conducted in accordance with the “Guide to Laboratory Animal Care and Use” (8th Edition) and approved by IACUC (Approval No.202410306). Female BALB/c-nu nude mice (45 days old) were purchased from Hubei Baitai Biotechnology Co., Ltd. Mice were housed in a pathogen-free environment with a 10 h light/14 h dark cycle and provided standard chow and water ad libitum.

#### Subcutaneous xenograft model

For the xenograft tumour model, the nude mice were randomly divided into four groups (*n* = 6 per group) based on animal ID numbers. ES2 cells were mixed with four kinds of THP-1 cells in a cell ratio of 1:1, and subcutaneously injected into the forelimbs of nude mice. Each mouse received 1 × 10^7^ cells in 100 μL of suspension. After four weeks of inoculation, tumour size was observed every four days and recorded.

### Molecular biology experiments (including WB, PCR, Co-IP, etc.)

#### WB

Primary antibodies were diluted at 1:1000 in TBST buffer containing 5% skim milk. Antibodies used included TREM2 (Abcam, ab318262), S100A9 (Proteintech, 26992-1-AP), IL-17Ra (Abcam, ab263908), IL-17 (Proteintech, 66148-1-Ig), CD206 (Proteintech, 60143-1-Ig), and Arg1 (AbclonBio, A4923).

#### qRT-PCR

Total RNA was extracted using commercial kits (ServiceBio, G3640-50T, G3333-50, G3326-01) according to the manufacturer’s instructions. qRT-PCR was performed using an ABI StepOnePlus system, and gene expression was calculated using the 2^−ΔΔCt^ method. Primer sequences are listed in Supplementary Table S1.

#### Flow cytometry

Antibodies used included CD11c (Proteintech, FITC-65086), CD11b (Proteintech, APC-65055), CD68 (Proteintech, PE-65187), CD206 (Proteintech, FITC-65155), Arg1 (Abclonal, A4923), and TREM2 (Proteintech, 27599-1-AP).

#### Cell proliferation assay (CCK-8)

Cells were cultured in 96-well plates at (8000 cells per well) and cultured overnight. After 24 h, 10 μL of CCK-8 solution (Beyotime, C0043) was added and incubated at 37 °C for 2 h. The absorbance at 450 nm was measured using a microplate reader.

#### Colony formation experiment

ES2 cells (2000 cells/well) were seeded into 6-well plates with three duplicate wells per group. Transwell chambers were placed, and THP-1-NC and THP-1-TREM2-OE cells were seeded in the upper chambers. Cultures were maintained at 37 °C with 5% CO₂ for 14 days, with THP-1-NC and THP-1-TREM2-OE cells refreshed every other day.

#### Wound healing assay

Cells were seeded into 6-well plates and cultured overnight. Linear scratches were generated using a 10 μL pipette tip, followed by replacement with fresh serum-free culture medium. The wound healing level was observed at 0 h and 24 h under the microscope.

#### Transwell migration assay

A total of 3 × 10^4^ cells in 200 μL serum-free medium were seeded to the upper chamber, while 800 μL of complete medium containing 30% FBS was added to the lower chamber. After incubation at 37 °C for 24 h, migrated cells were fixed, stained with crystal violet, and quantified microscopically.

#### Immunofluorescence staining

Cells were fixed with 4% paraformaldehyde for 15 min, permeabilized with 0.1% Triton X-100 at room temperature for 10 min, and blocked with 3% BSA at room temperature for 30 min. After incubating with primary antibody at 4 °C overnight, the cells were washed three times in PBS and incubated with secondary antibody at 37 °C in the dark for 1 h. DAPI staining (Servicebio, G1407) was performed before mounting, followed by imaging using a microscope (LIFE EVOS).

#### EdU proliferation assay

Cells were incubated with 10 μM EdU (Beyotime, C0075L) for 2 h at 37 °C, fixed, permeabilized, and stained according to the manufacturer’s instructions. Images were acquired using a 20× fluorescence microscope.

#### Co-immunoprecipitation (Co-IP)

Cell lysates were prepared under ice-cold conditions and incubated with Protein A/G magnetic beads (Selleck, B23201). Samples were immunoprecipitated using anti-TREM2 antibody overnight at 4 °C, followed by bead incubation, washing, SDS-PAGE separation, and immunoblot analysis.

#### Hematoxylin and eosin (H&E) staining and immunohistochemistry (IHC)

Tumour tissues were fixed in 10% formalin, embedded in paraffin, sectioned, and stained following standard H&E protocols. For IHC, primary antibodies against CD45, CD68, TREM2, CD206, and Arg1 were applied, followed by HRP-conjugated secondary antibodies and DAB visualisation.

### Statistical analysis

Data were obtained from at least three (*n* = 3) independent experiments and analysed using GraphPad Prism 9 (GraphPad Software, San Diego, CA). Results are presented as mean ± standard deviation (SD). Statistical significance was assessed using unpaired *t*-test and one-way ANOVA, with *P* < 0.05 considered statistically significant (*P < 0.05, **P < 0.01, ***P < 0.001, ****P < 0.0001, ns not significant).

## Supplementary information

Supplementary figures and table

wb supplementary

## Data Availability

The data that support the findings of this study are available on request from the corresponding author.
