# Supplementary material for: Discovery of a non-canonical TREM2-S100A9 axis that drives pro-tumorigenic macrophage polarization and sensitizes ovarian clear cell carcinoma to IL-17 blockade
Source: Cell Death Discov. 2026 Jul 7;12:388. doi: 10.1038/s41420-026-03244-2 (PMC13623968; doi:10.1038/s41420-026-03244-2)
Supplement: Supplementary file 1 [file 41420_2026_3244_MOESM2_ESM.pdf]

Figure2C

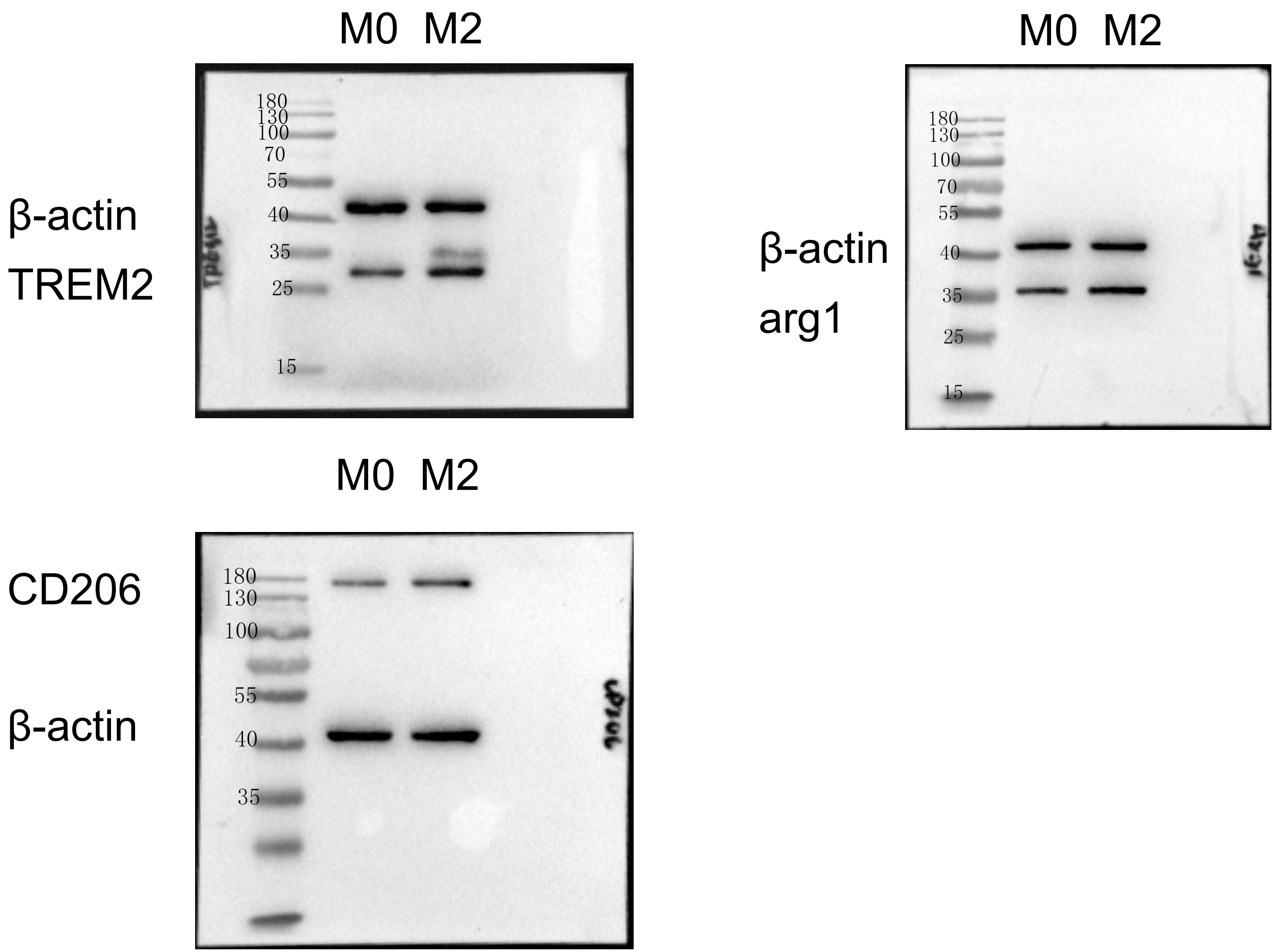

M0 M2
β-actin
TREM2
180
130
100
70
55
40
35
25
15
M0 M2
β-actin
arg1
180
130
100
70
55
40
35
25
15
M0 M2
CD206
β-actin
180
130
100
55
40
35

Figure3C

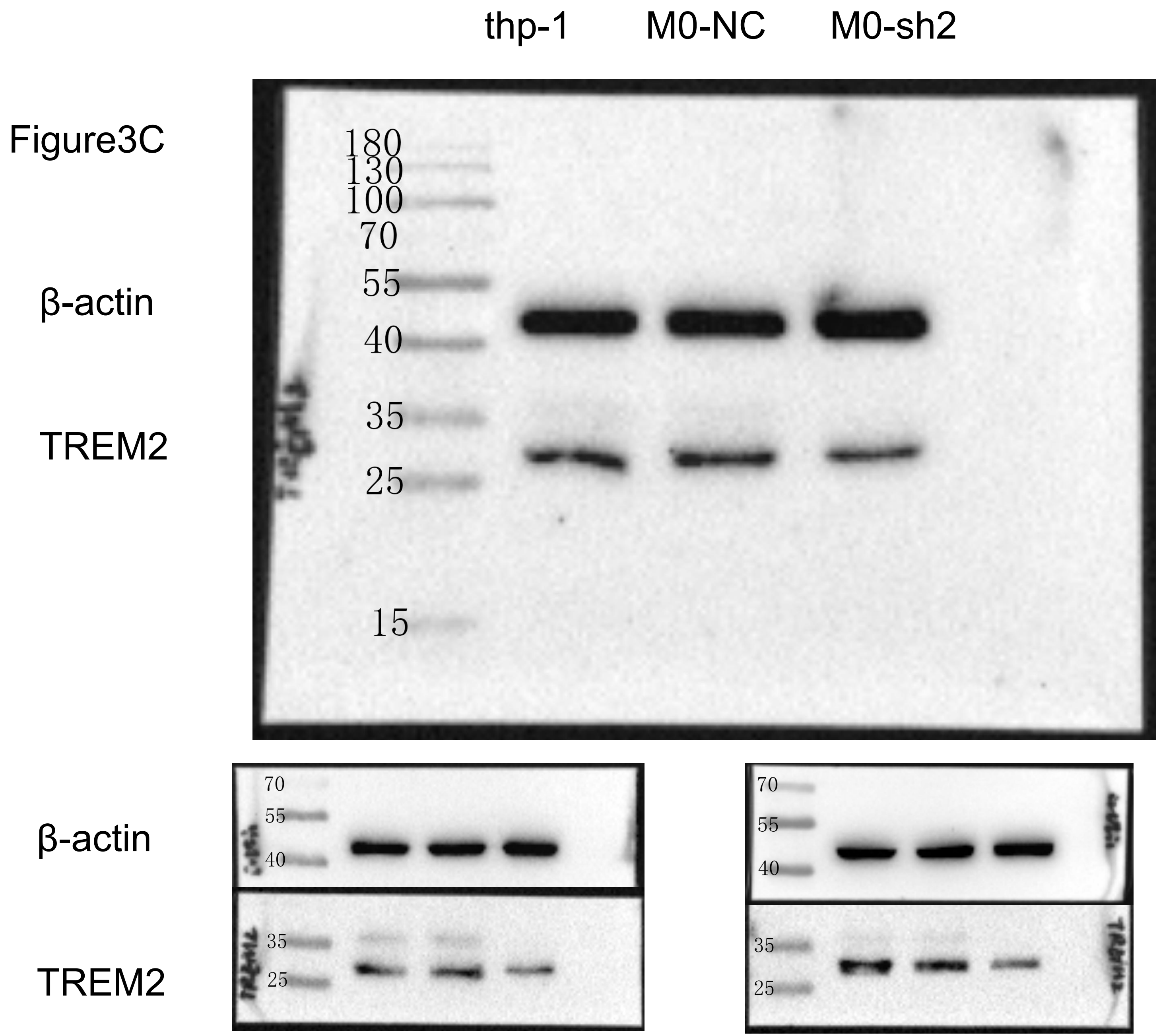

thp-1
M0-NC
M0-sh2
β-actin
TREM2
180
130
100
70
55
40
35
25
15
β-actin
TREM2
70
55
40
35
25
70
55
40
35
25

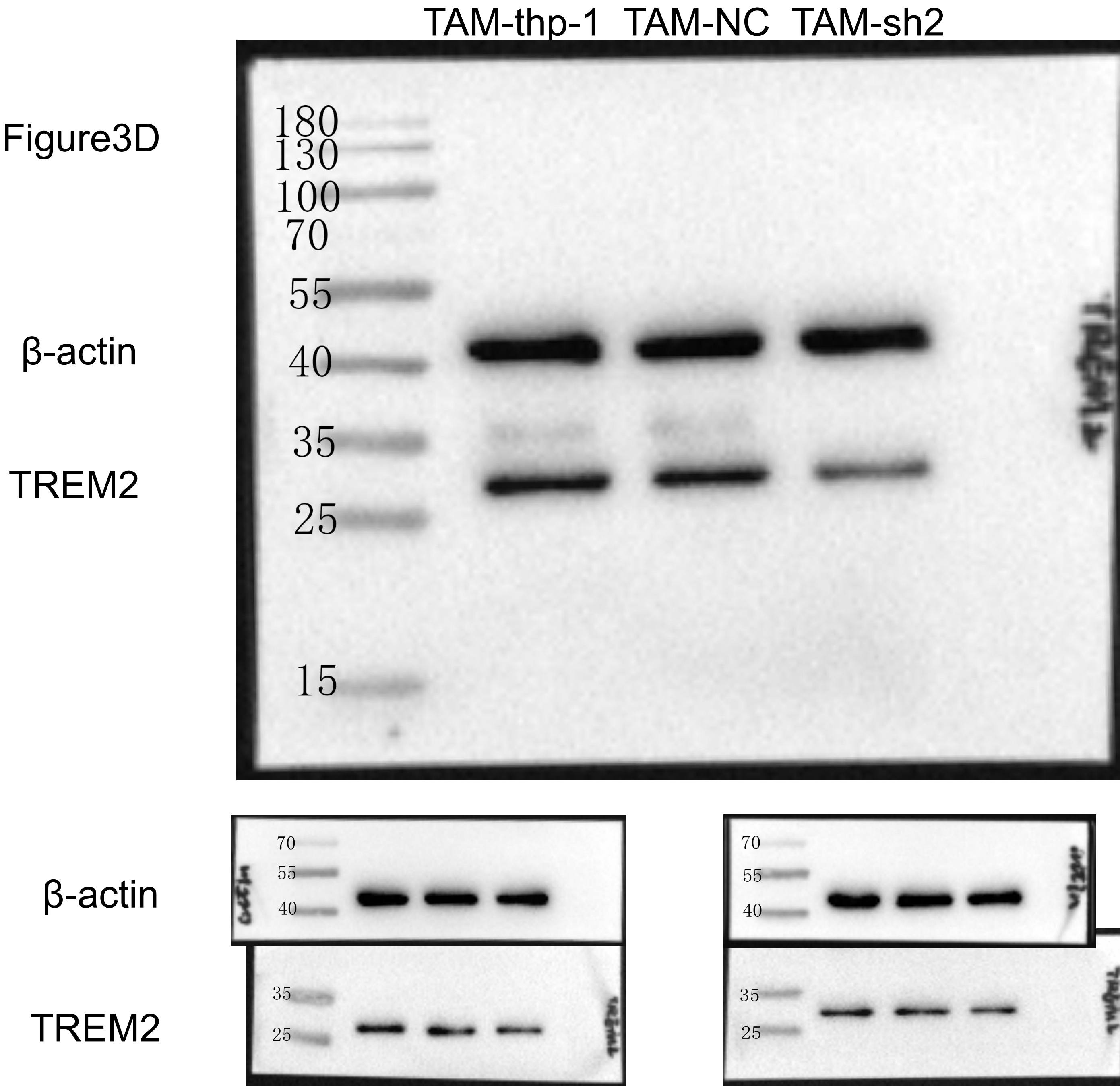
TAM-thp-1 TAM-NC TAM-sh2
Figure3D
β-actin
TREM2
180
130
100
70
55
40
35
25
15
β-actin
TREM2
70
55
40
35
25
70
55
40
35
25

Figure3F

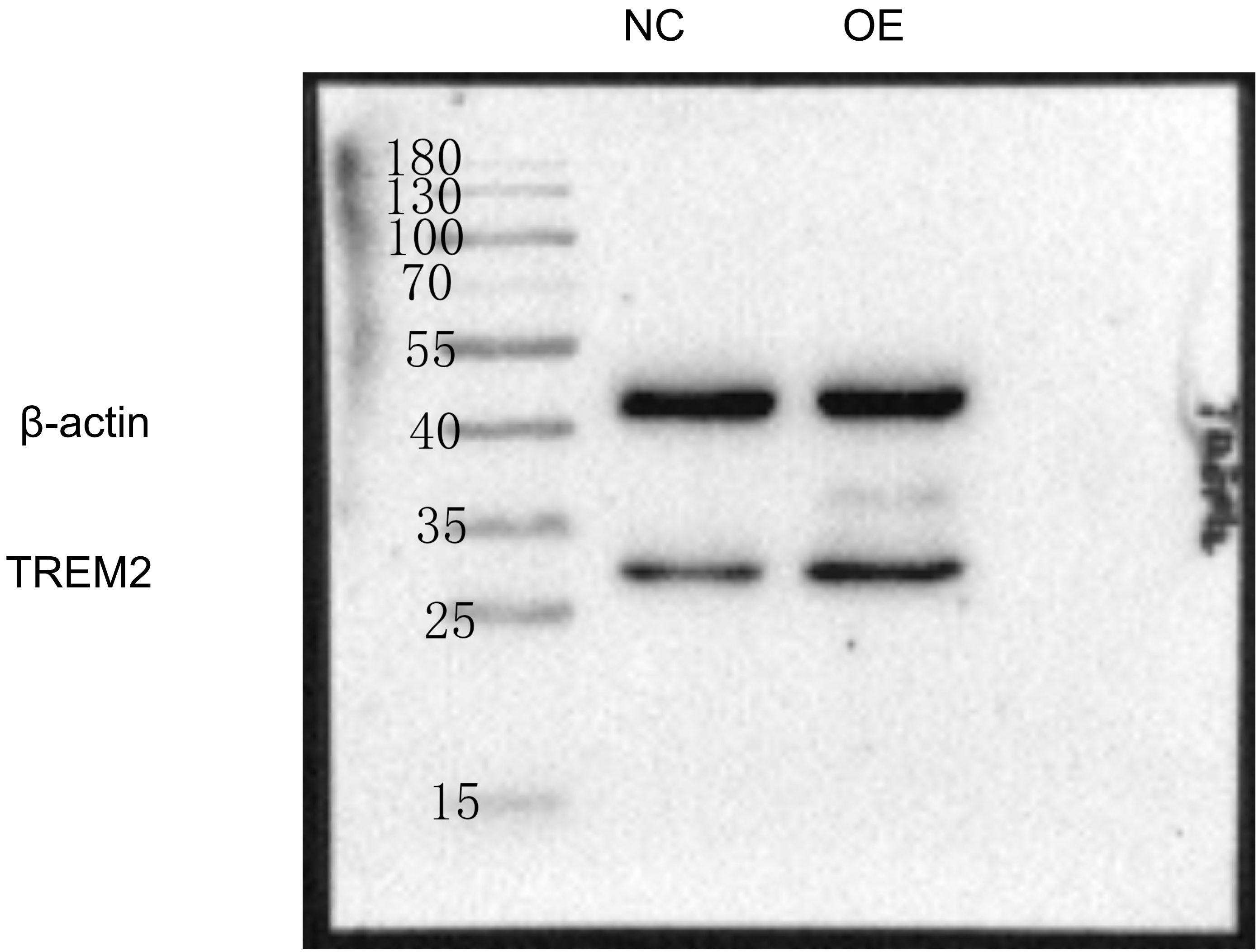

Figure3G

ES2+NC ES2+OE

β-actin

TREM2

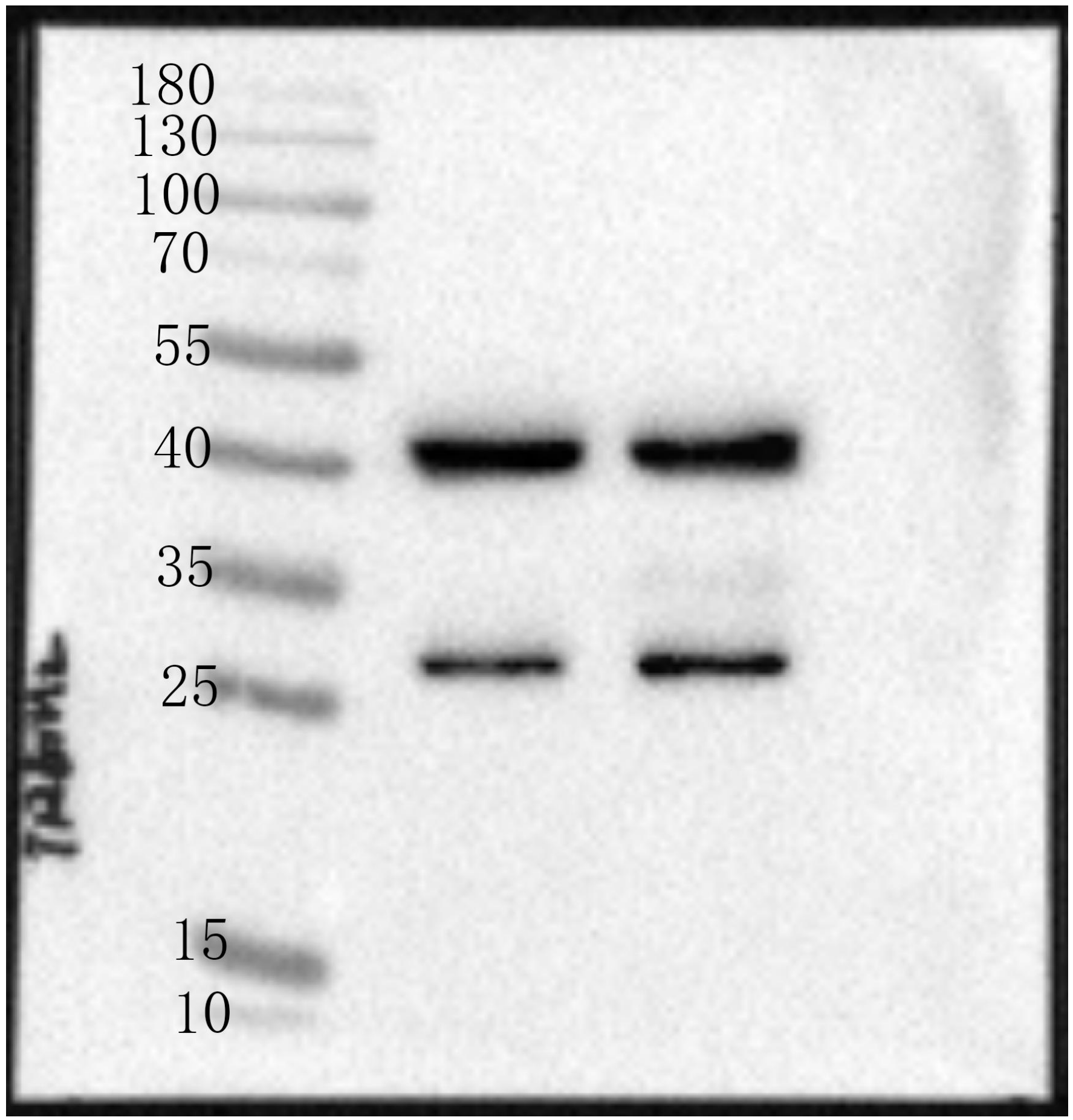

Figure4F

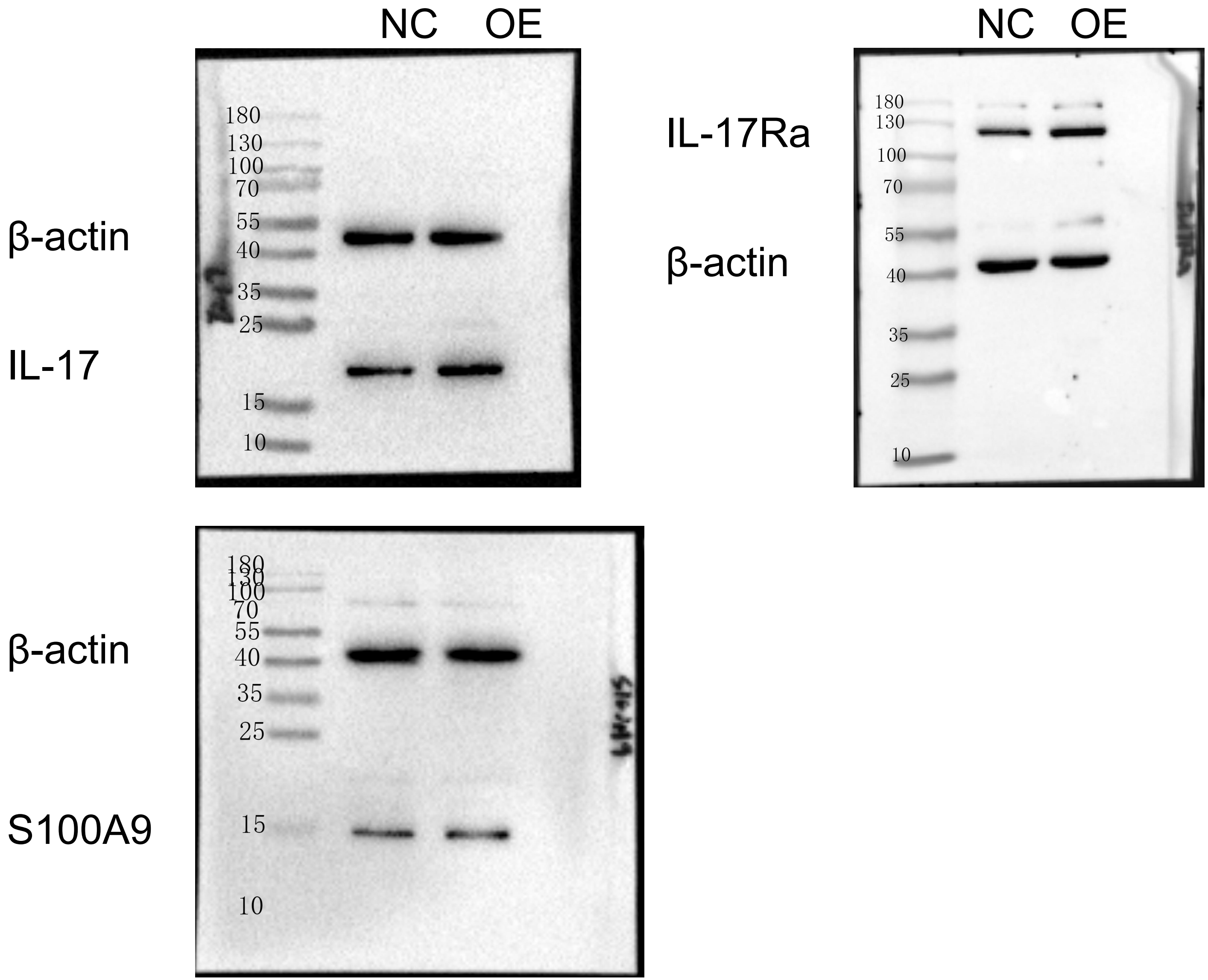
NC
OE
β-actin
IL-17
180
130
100
70
55
40
35
25
15
10
NC
OE
IL-17Ra
β-actin
180
130
100
70
55
40
35
25
10
β-actin
S100A9
180
130
100
70
55
40
35
25
15
10

Figure5A

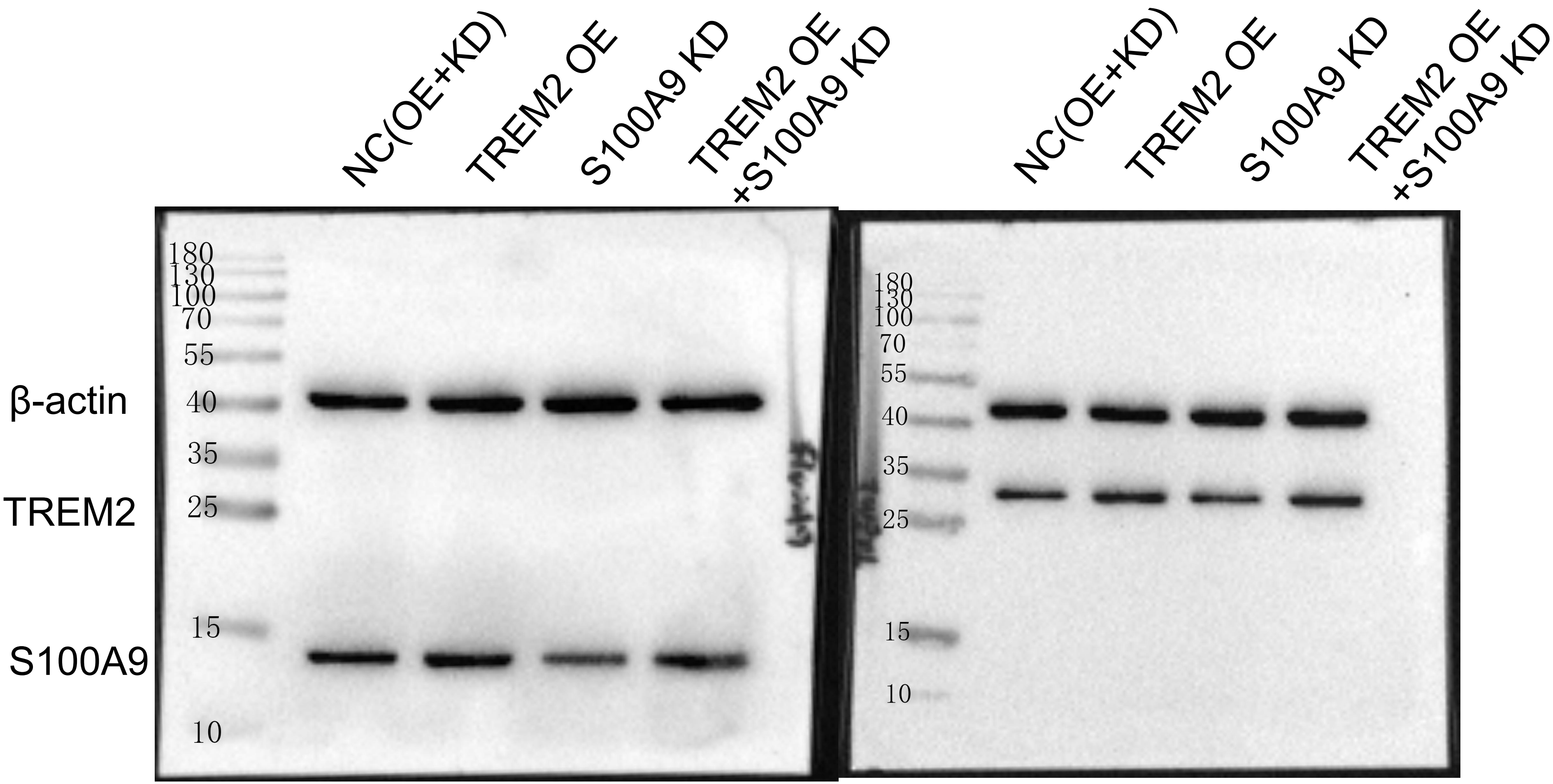
NC(OE+KD)
TREM2 OE
S100A9 KD
TREM2 OE
+S100A9 KD
NC(OE+KD)
TREM2 OE
S100A9 KD
TREM2 OE
+S100A9 KD
β-actin
TREM2
S100A9
180
130
100
70
55
40
35
25
15
10
180
130
100
70
55
40
35
25
15
10

Figure6C

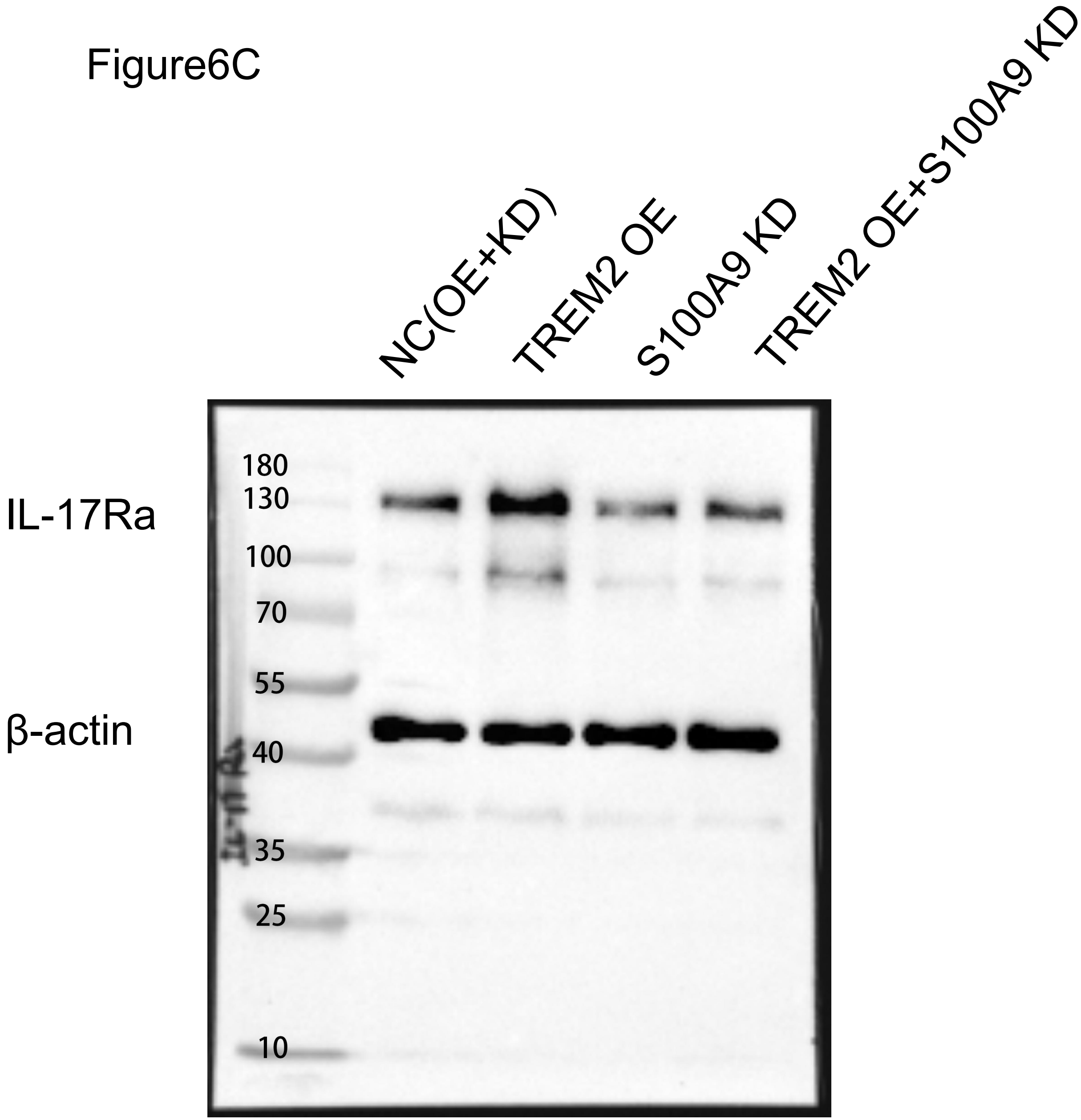

NC(OE+KD)
TREM2 OE
S100A9 KD
TREM2 OE+S100A9 KD
IL-17Ra
β-actin
180
130
100
70
55
40
35
25
10

Figure6D

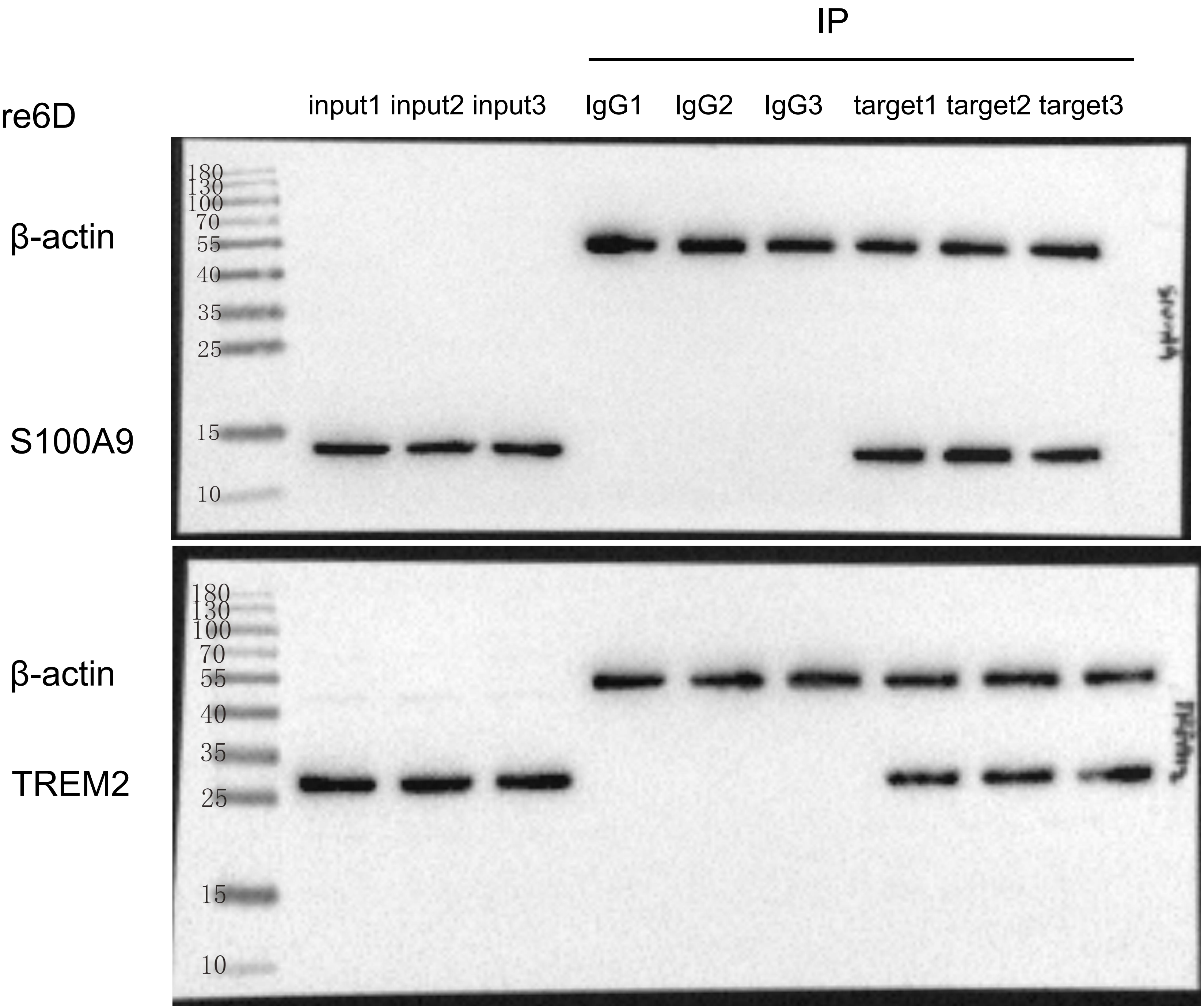

IP
input1 input2 input3
IgG1
IgG2
IgG3
target1 target2 target3
β-actin
S100A9
β-actin
TREM2
180
130
100
70
55
40
35
25
15
10

Figure7B

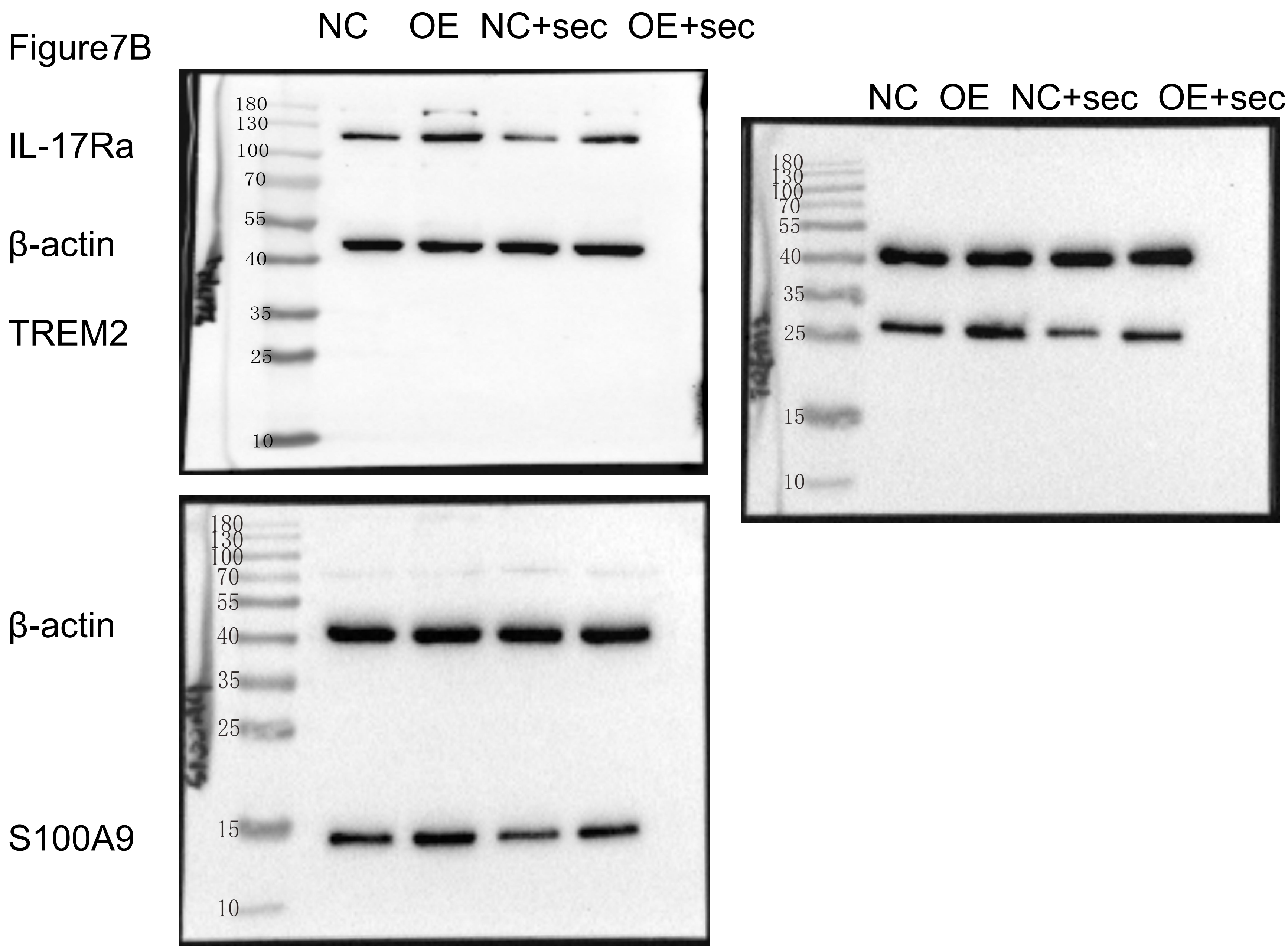
NC
OE
NC+sec
OE+sec
IL-17Ra
β-actin
TREM2
180
130
100
70
55
40
35
25
10
NC
OE
NC+sec
OE+sec
180
130
100
70
55
40
35
25
15
10
β-actin
S100A9
180
130
100
70
55
40
35
25
15
10

Figure8D

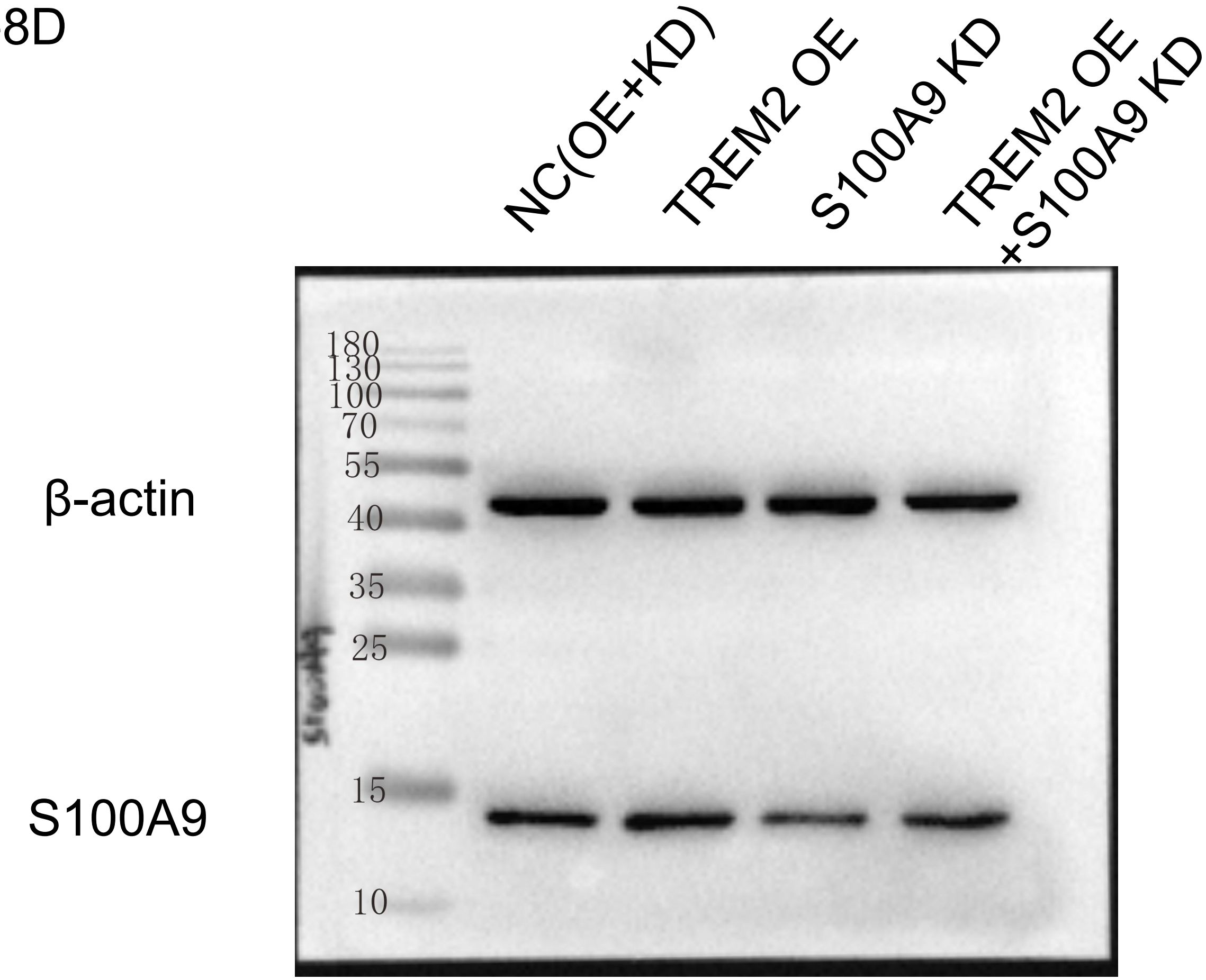
NC(OE+KD)
TREM2 OE
S100A9 KD
TREM2 OE
+S100A9 KD
180
130
100
70
55
40
35
25
15
10
β-actin
S100A9

Figure8D

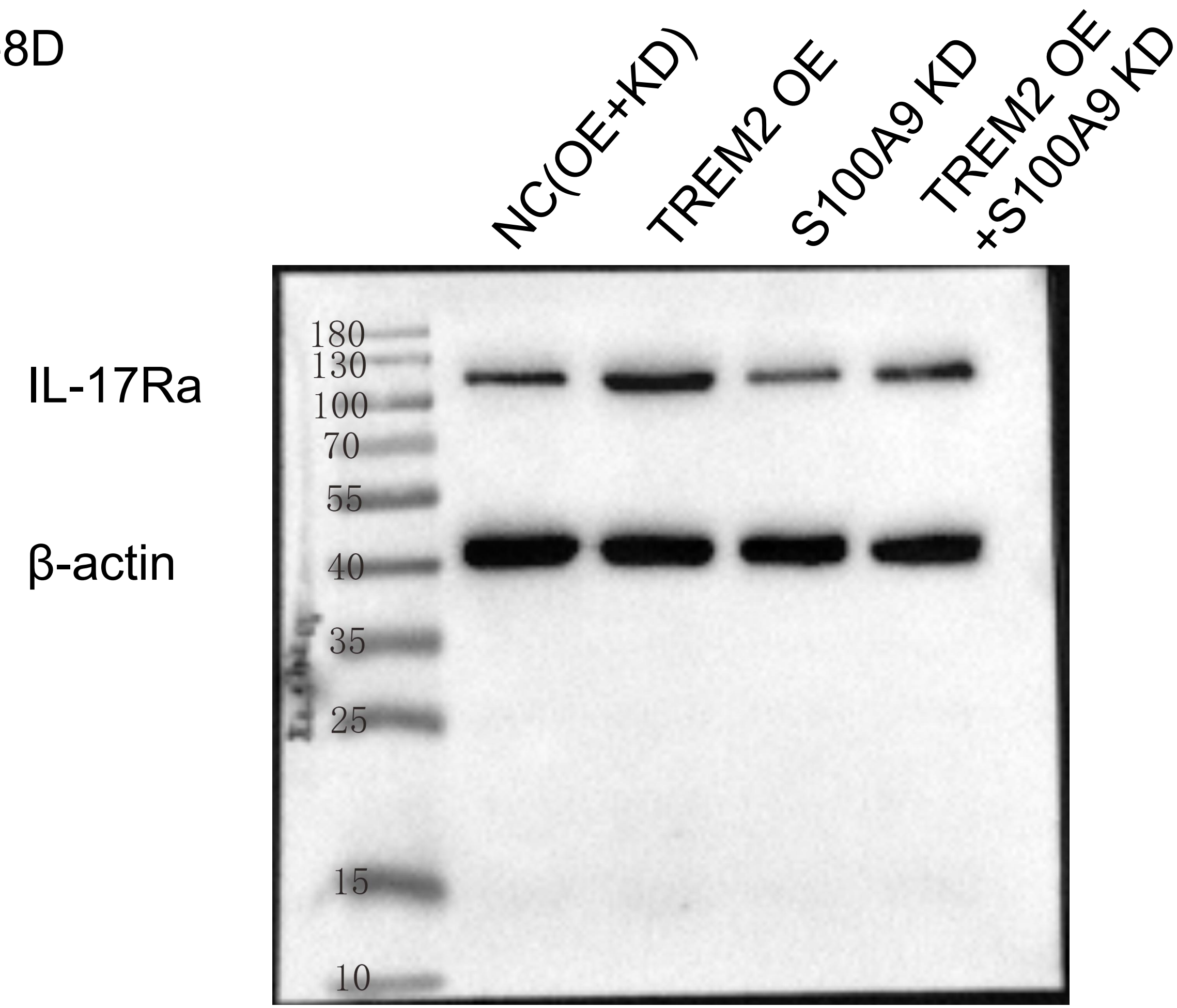
NC(OE+KD)
TREM2 OE
S100A9 KD
TREM2 OE
+S100A9 KD
IL-17Ra
β-actin
180
130
100
70
55
40
35
25
15
10

Figure8D

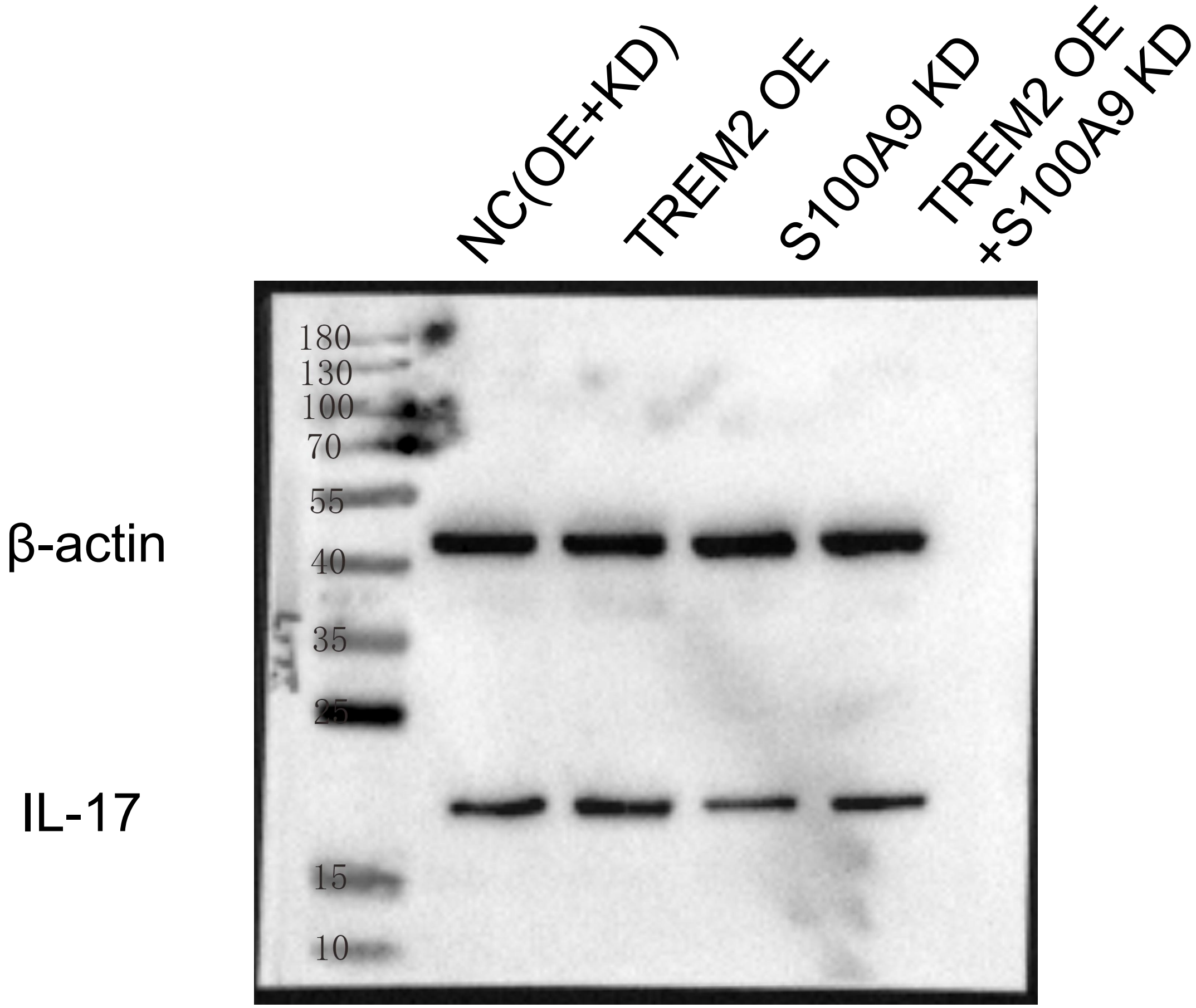
NC(OE+KD)
TREM2 OE
S100A9 KD
TREM2 OE
+S100A9 KD
β-actin
IL-17
180
130
100
70
55
40
35
25
15
10

Figure8D

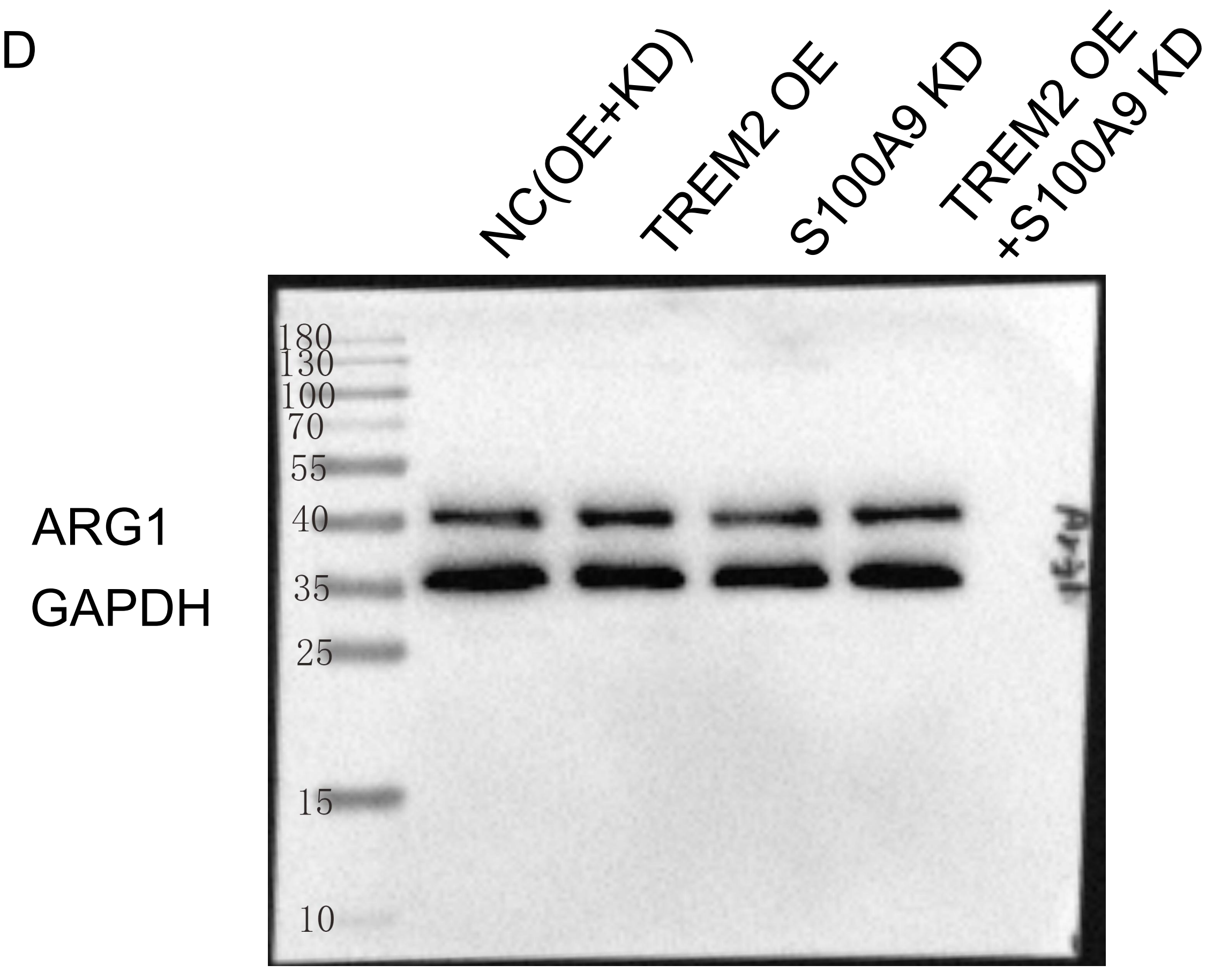

NC(OE+KD)
TREM2 OE
S100A9 KD
TREM2 OE
+S100A9 KD
180
130
100
70
55
40
35
25
15
10
ARG1
GAPDH

Figure8D

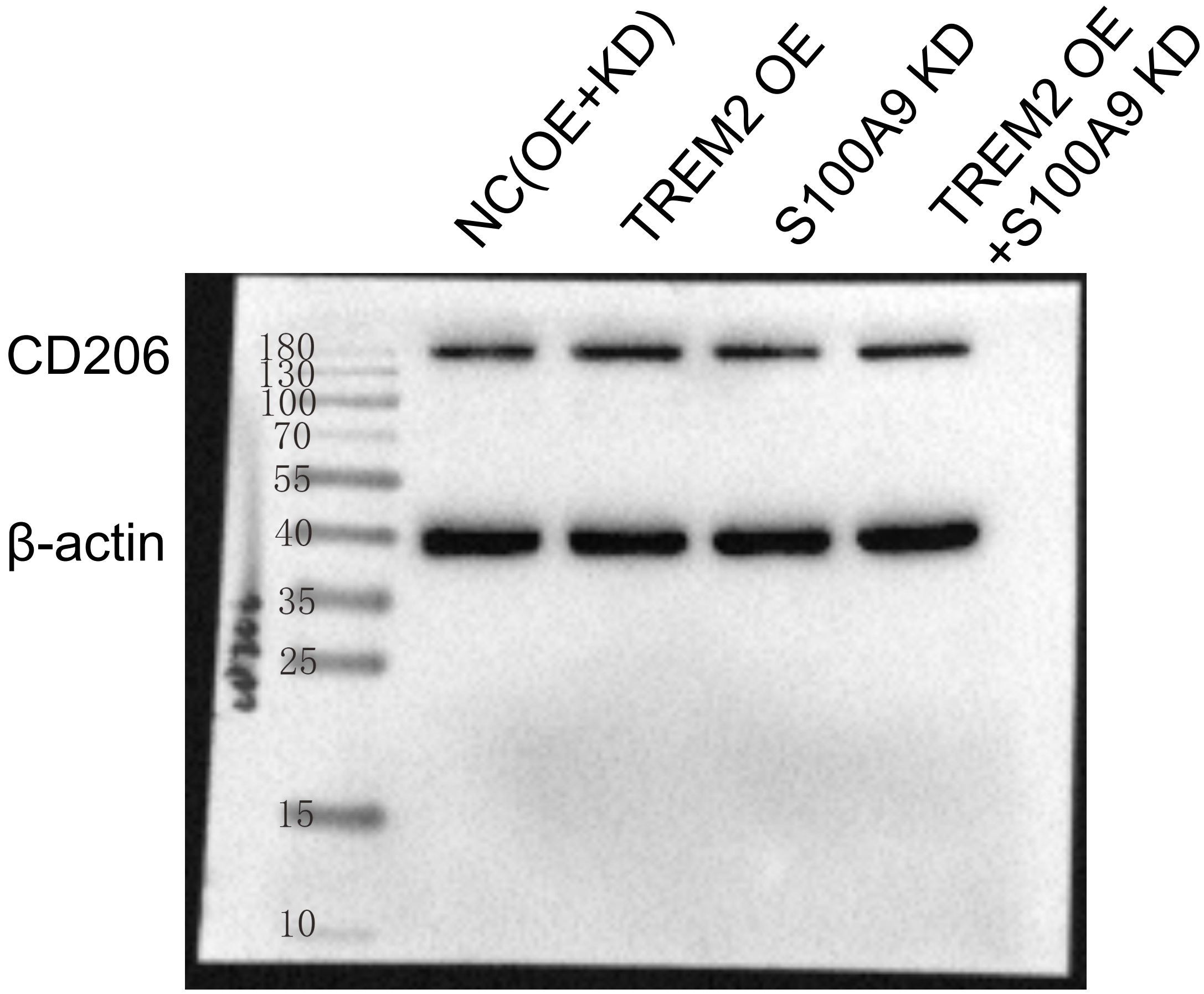

NC(OE+KD)
TREM2 OE
S100A9 KD
TREM2 OE
+S100A9 KD
CD206
β-actin
180
130
100
70
55
40
35
25
15
10
