# Supplementary material for: Discovery of a non-canonical TREM2-S100A9 axis that drives pro-tumorigenic macrophage polarization and sensitizes ovarian clear cell carcinoma to IL-17 blockade
Source: Cell Death Discov. 2026 Jul 7;12:388. doi: 10.1038/s41420-026-03244-2 (PMC13623968; doi:10.1038/s41420-026-03244-2)
Supplement: Supplementary file 2 [file 41420_2026_3244_MOESM1_ESM.docx]

Supplementary Figure 1 Tissue microarray (figure 1D-E)

Immunofluorescence of TMA

Immunohistochemistry of TMA

Supplementary Figure 2 (figure2B)

Representative results of the flow cytometric analysis of macrophage differentiation markers. M0 macrophages treated with PMA were subjected to flow cytometry to detect the expression of macrophage differentiation markers, including CD11c, CD11b, and CD68.

Supplementary Figure 3 (figure2F)

Representative results of the flow cytometric detection of TREM2, CD206, and ARG1 expression. M0 macrophages treated with PMA, M2 macrophages treated with OC tumor condition medium.

Supplementary Figure 4 (figure3E)

TAM-NC, TAM-SH2, TAM-THP-1 cells were induced to differentiate into macrophages by PMA and then treated with ovarian cancer cell-derived conditioned medium. The expression levels of CD206 and Arg1 were detected by flow cytometry. Representative results were presented.

Supplementary Figure 5 (figure3M)

Analysis of cell cycle distribution by flow cytometry. Representative results were presented.

**Supplementary Table S1** Primer sequences for RT-qPCR.

| Name | Primer | Sequence5'-3' |
| --- | --- | --- |
| TREM2 | Forward | GGTCAGCACGCACAACTTG |
|  | Reverse | CTCGAAGCTCTCAGACTCCC |
| Arg1 | Forward | TGGACAGACTAGGAATTGGCA |
|  | Reverse | CCAGTCCGTCAACATCAAAACT |
| CD206 | Forward | GGGTTGCTATCACTCTCTATGC |
|  | Reverse | TTTCTTGTCTGTTGCCGTAGTT |
| GAPDH | Forward | CTGGGCTACACTGAGCACC |
|  | Reverse | AAGTGGTCGTTGAGGGCAATG |
| S100A8 | Forward | ATGCCGTCTACAGGGATGAC |
|  | Reverse | ACTGAGGACACTCGGTCTCTA |
| S100A9 | Forward | GGTCATAGAACACATCATGGAGG |
|  | Reverse | GGCCTGGCTTATGGTGGTG |
| CXCL8 | Forward | TTTTGCCAAGGAGTGCTAAAGA |
|  | Reverse | AACCCTCTGCACCCAGTTTTC |
| LTC4S | Forward | GTCCTGCTGCAAGCCTACTT |
|  | Reverse | GTCCTGCTGCAAGCCTACTT |
| PDE9A | Forward | GACTCCTCGACGCGATGTTC |
|  | Reverse | TTTCTGTAGTTGTCGTGGACG |
| Β-ACTIN | Forward | CATGTACGTTGCTATCCAGGC |
|  | Reverse | CTCCTTAATGTCACGCACGAT |
